# Mapping Genetic Modifiers of Polyp Formation in *Smad4*-Deficient Juvenile Polyposis Using the Collaborative Cross Mouse Population

**DOI:** 10.3390/cells15100853

**Published:** 2026-05-07

**Authors:** Osayd Zohud, Kreem Midlej, Iqbal M. Lone, Aysar Nashef, Imad Abu-Elnaaj, Fuad A. Iraqi

**Affiliations:** 1 Department of Clinical Microbiology and Immunology, Faculty of Medicine and Health Sciences, Tel-Aviv University, Tel-Aviv 6997801, Israel; osaydzohud@mail.tau.ac.il (O.Z.); kareemmidlej@mail.tau.ac.il (K.M.); iqbalzoo84@gmail.com (I.M.L.); 2 Department of Oral and Maxillofacial Surgery, Baruch Padeh Medical Center, Poriya 1528001, Israel; dr.aysarn@gmail.com (A.N.); iabu@poria.health.gov.il (I.A.-E.); 3 Azrieli Faculty of Medicine, Bar-Ilan University, Safed 1311502, Israel; 4 Department of Oral and Maxillofacial Surgery, Meir Medical Center, Kfar Saba Affiliated to the Faculty of Medicine and Health Sciences, Tel-Aviv University, Kfar Saba 4428164, Israel

**Keywords:** juvenile polyposis syndrome, *Smad4* knockout, collaborative cross mice, quantitative trait loci, genetic modifiers, colorectal cancer, intestinal polyps

## Abstract

**Highlights:**

**What are the main findings?**

- Novel genetic modifiers of intestinal polyp susceptibility were identified using Collaborative Cross mice carrying Smad4 mutations.
- Multiple QTL influencing polyp burden were detected across chromosomes 12, 14, and 16, including sex-specific loci.

**What are the implications of the main findings?**

- Genetic background significantly influences phenotypic heterogeneity in Juvenile Polyposis Syndrome.
- Identified candidate genes provide potential targets for mechanistic and translational studies.

**Abstract:**

Juvenile Polyposis Syndrome (JPS) is an autosomal dominant disorder characterized by multiple gastrointestinal polyps and an increased risk of cancer, most commonly associated with mutations in the tumor suppressor gene *Smad4*. However, substantial phenotypic variability exists among individuals carrying identical mutations, suggesting the presence of genetic modifiers. In this study, we used the genetically diverse Collaborative Cross (CC) mouse population crossed with Smad4 knockout mice to identify loci influencing intestinal polyp development. A cohort of 260 F1 mice derived from 14 CC lines was assessed for polyp number and size across intestinal segments. Quantitative trait locus (QTL) mapping revealed several significant loci, including regions on chromosomes 16, 14, and 12, which were designated *Ipsl*1, *Ipsl*2, and *Ipsl*3 for Intestinal Polyposis Susceptibility locus (*Ipsl*), respectively, in the full population, as well as additional sex-specific loci in male and female cohorts. Pathway enrichment analysis of genes within these regions highlighted functional associations with immune signaling, ubiquitin–proteasome degradation, and metabolic regulation. Candidate genes, including *STAM2*, *PSMD6*, *NAMPT*, and *CACNB4*, emerged as potential modifiers of polyp susceptibility. These findings highlight the complex genetic architecture underlying JPS phenotypes and provide candidate loci for future functional and translational investigations.

## 1. Introduction

Juvenile Polyposis Syndrome (JPS) is an autosomal dominant disorder characterized by multiple juvenile polyps in the gastrointestinal tract and a heightened risk of gastrointestinal cancers [1,2]. Mutations in tumor suppressor genes such as *Smad4* and *BMPR1A* are central to its pathogenesis, with *Smad4* being the most commonly affected [3]. Occasionally, *PTEN* mutations have also been implicated, underscoring the genetic complexity of JPS [4].

Clinically, JPS presents variable, even among individuals carrying the same mutation. Diagnostic criteria include five or more juvenile polyps throughout the gastrointestinal tract or any number of juvenile polyps with a family history of the syndrome [5]. While most polyps are benign, they carry a significant risk of malignant transformation if untreated [6]. This variation in disease severity—despite shared mutations—suggests the involvement of genetic modifiers [7].

Colorectal cancer (CRC), the second leading cause of cancer-related deaths globally, shares many genetic and epigenetic underpinnings with JPS [8,9]. Key signaling pathways such as Wnt, TGF-β, Hedgehog, and Notch play vital roles in both JPS and CRC progression [10,11,12,13].

Mouse models have been instrumental in elucidating the molecular mechanisms underlying JPS and its associated pathways. Genetically engineered mice with mutations in *Smad4*, *BMPR1A*, or *PTEN* exhibit polyp formation and have been used to study the role of genetic modifiers [14,15,16,17]. Such modifiers may alter disease severity or onset and are often challenging to detect in human GWAS due to sample size constraints [18]. In contrast, mouse models allow controlled breeding and targeted mutation, making them ideal for modifier discovery [18].

A powerful resource for such research is the Collaborative Cross (CC) mouse panel—derived from eight diverse founder strains, including classical laboratory and wild-derived lines [19]. The CC population captures a wide spectrum of genetic diversity comparable to human populations [20], and CC-derived offspring have been shown to uncover novel modifiers in polyposis and cancer models [18,21,22]. While previous CC-based studies have primarily focused on *Apc*-driven models of intestinal tumorigenesis, the genetic modifiers of polyp development in a *Smad4*-deficient context remain less well characterized.

Smad4-deficient mice (C57BL/6J-*Smad4*^tm1Mak^) recapitulate several features of human JPS, including the formation of intestinal polyps [23]. However, variation in polyp burden even among littermates implies the action of background modifiers. Understanding these modifiers could clarify why clinical severity varies among JPS patients sharing the same germline mutations and may lead to new therapeutic targets or risk-stratification tools.

In this study, we aimed to uncover genetic modifiers influencing polyp development in a *Smad4*-deficient background by crossing CC mice with *Smad4* knockout mice. We performed quantitative trait locus (QTL) mapping across 260 F1 progeny from 14 CC lines to identify novel loci affecting polyp number, size, and distribution across intestinal segments. Our working hypothesis was that the genetic heterogeneity introduced by the CC lines would reveal novel modifier loci modulating the phenotypic expression of *Smad4* loss.

## 2. Materials and Methods

### 2.1. Ethics and Animal Welfare Considerations

This research adhered to the national guidelines for the ethical treatment of laboratory animals. The study’s protocol received approval from the Institutional Animal Care and Use Committee (IACUC) of Tel Aviv University, under the authorization number (01-19-044). Daily health monitoring of the mice was conducted, with specific criteria set for humane euthanasia based on weight loss or observed distress, in consultation with the facility’s Veterinary staff.

### 2.2. CC Lines Crossbreeding to Generate F1 Offspring

The detailed process of developing these Collaborative Cross (CC) lines has been previously described [20,21]. The study cohort included 260 mice (129 males and 131 females) derived from 14 distinct CC lines. The mice, aged 18–20 weeks, were supplied by the Small Animal Facility at the Sackler Faculty of Medicine, Tel Aviv University (TAU). They were housed in open-top cages with hardwood chip bedding, under a 12:12-h light cycle, and maintained at a temperature of 21–23 °C. The mice had ad libitum access to tap water and a standard rodent chow diet, which provided 18% of total calories from fat, 24% from protein, and 58% from carbohydrates (TD.2018SC; Teklad Global, Harlan, Madison, WI, USA), starting from weaning at 3 weeks of age until they reached 20 weeks. CC lines’ high molecular genomic DNA was genotyped using MDA, MUGA, and MegaMuga SNP arrays. The genotype database is publicly available [https://www.jax.org] (accessed on 15 December 2025). The C57BL/6 J-*Smad4*^tm1Mak^ strain was sourced from the Jackson Laboratory (Bar Harbor, ME, USA). Crosses between female mice from available CC strains and C57BL/6 J-*Smad4*^tm1Mak^ males resulted in F1 offspring. Genotypic screening for the Smad4 gene among these produced F1 mice across lines for inclusion in subsequent experiments, as detailed in [17].

### 2.3. Genomic DNA Extraction and Genotyping of F1 Mice

For genomic DNA extraction, we utilized the NaOH method as described in [17,24]. Tail samples measuring 3–4 mm were placed in Eppendorf tubes, and 75 µL of 25 mM NaOH and 0.2 mM EDTA was added. The samples were heated at 98 °C for 1 h on a heating block and then cooled on ice. After heating, the samples were neutralized with 75 µL of 40 mM Tris-HCl (pH 5.5). The mixture was centrifuged at 4000 rpm for 3 min to clarify the samples, which were subsequently ready for PCR analysis. The PCR genotyping protocol utilized specific primer sets for the *Smad4* gene. Reaction A amplified a 200 bp segment from the wild-type *Smad4* gene, while Reaction B generated a 300 bp product indicative of the knockout *Smad4* genotype. Genotyping involved 30 cycles of denaturation, annealing, and extension. Genotyping of F1 mice was described earlier [25].

### 2.4. Tissue Collection

At 80 weeks, mice were terminated using CO_2_. Small intestine and colon samples were collected and washed with Phosphate-Buffered Saline. Intestines were categorized into segments (SB1: proximal; SB2: middle; and SB3: distal) and analyzed for polyp counts [17].

### 2.5. Intestine Whole Mounts and Polyp Assessment

Intestines were fixed in 10% Neutral Buffered Formalin for 24 h and then stained with methylene blue as described earlier [18]. Polyps were counted and categorized based on their diameter into three groups: A polyps (more than 3 mm), B polyps (1–3 mm), and C polyps (less than 1 mm).

### 2.6. Data Analysis

Initial statistical analyses were performed using SPSS (version 19.0, IBM Corp., Armonk, NY, USA). ANOVA was used to assess variation in total polyp counts among different CC-B/6-Min crosses, as described previously [17].

### 2.7. Reconstruction of CC Ancestral Genome Mosaics

SNPs were mapped onto the mouse genome, and CC ancestral genome mosaics were reconstructed using R-QTL. Locus-specific fractions of CC lines carrying each founder were estimated.

### 2.8. QTL Analysis

QTL mapping was performed using R/qtl2 (version 0.3) within the R statistical environment (version 4.2.x). Genotype probabilities were calculated using a hidden Markov model, and genome scans were conducted using a linear mixed model framework to account for population structure.

Significance thresholds for QTL detection were determined using permutation testing (10,000 permutations), and genome-wide significance levels were defined based on empirical LOD thresholds. Confidence intervals were estimated using 95% Bayesian credible intervals, as described earlier [26].

QTL mapping was conducted separately for the whole F1 population, as well as for male-only (n = 129) and female-only (n = 131) subgroups, to identify both shared and sex-specific loci. The same statistical framework, permutation testing, and Bayesian credible interval settings were applied across all analyses.

### 2.9. Estimation of QTL Confidence Intervals and Founder Effects

Confidence intervals were estimated through simulation, considering local patterns of linkage disequilibrium. Founder strain effects at each QTL were compared to WSB/EiJ using merge analysis. Variants were classified based on genome annotation.

### 2.10. List of Suggested Candidate Genes

The SNP tools package and MGI database identified candidate genes within QTL intervals. Protein-coding genes and non-coding RNA genes were considered.

### 2.11. Pathway Enrichment Analysis

Genes identified within significant QTL intervals were analyzed for pathway enrichment to elucidate potential functional mechanisms. The QTL regions spanned chromosomes 2, 12, and 14 in mice and were identified using significance thresholds of *p* < 0.05 and *p* < 0.1, based on association statistics. For each region, genes were extracted based on their Ensemble gene IDs and chromosomal coordinates. These gene sets were then subjected to functional enrichment analysis using the *g:Profiler* (accessed: August 2025) and *Enrichr* (accessed: August 2025) platforms, default parameters were used unless otherwise specified. [27,28]. The enrichment focused on three major pathway databases: WikiPathways [29], CORUM [30], and Reactome [31]. Statistical significance was assessed using hypergeometric testing and adjusted for multiple comparisons using the Benjamini–Hochberg false discovery rate (FDR). Pathways with an FDR-adjusted *p*-value < 0.1 were considered biologically relevant. Enrichment statistics such as overlap count, odds ratio, and combined score were used to rank pathways of interest.

### 2.12. Gene Functional Analysis

To provide a deeper biological context for the genes identified in QTL intervals, we performed manual gene function annotation and literature-based validation. Each gene identified through pathway enrichment was cross-referenced with multiple publicly available databases, including UniProt, GeneCards, Mouse Genome Informatics (MGI), and NCBI Gene. These resources were used to gather information on molecular function, biological processes, tissue specificity, and phenotypic associations in murine models.

Genes that appeared in multiple pathways or complexes (e.g., PSMD6, *STAM*2, *NAMPT*) were further prioritized for literature review. Manual curation involved searching PubMed using combinations of gene names and keywords related to the observed trait categories (e.g., “*PSMD*6 + proteasome + mouse,” “*STAM*2 + endocytosis + signaling,” “*CACNB*4 + neurodevelopment + epilepsy”). Preference was given to studies in mouse models or those directly linking the gene to phenotypes analogous to the QTL traits.

In addition, for genes involved in critical pathways such as ubiquitin-mediated degradation, neurophysiology, or immune signaling, supporting publications were cited directly to reinforce biological plausibility. This integrative approach allowed us to link statistical enrichment results with known gene function, providing a biologically informed interpretation of the QTL landscape.

### 2.13. Network and Visualization Analysis

To explore gene–pathway associations and identify potential regulatory hubs, we constructed bipartite networks linking QTL-associated genes to enriched pathways. Analyses were conducted separately for WikiPathways (2024, Mouse), Gene Ontology (Biological Process, 2025), and in a combined context.

Hub genes were defined as those present in two or more enriched pathways among the top 30 pathways ranked by enrichment *p*-value were selected to balance biological interpretability and network complexity. This threshold was not based on a fixed statistical cutoff but was chosen to focus on the most relevant signals while maintaining clarity of visualization. Genes and pathway terms were used to generate bipartite networks using the NetworkX Python package (version 2.8.4) in Python (version 3.8.10). Pathway nodes were visualized as gray squares and gene nodes as blue circles, with edges indicating gene membership in each pathway. Node layouts were computed using a force-directed spring algorithm with default parameters to optimize clarity and separation. Pathways not connected to any gene were excluded from the final graphs to enhance interpretability.

### 2.14. Gene × Pathway Heatmap

To complement network visualizations, we constructed a binary heatmap of gene–pathway membership using the top-enriched WikiPathways (Mouse). Genes were listed in rows, and pathways in columns. Cells were shaded blue if the gene was annotated in the corresponding pathway (1 = present). Only pathways with at least two gene annotations were retained. Heatmaps were generated in Python using the Seaborn library.

### 2.15. Transcription Factor Enrichment

In addition to pathway and ontology analyses, we tested whether QTL candidate genes were enriched for transcription factor (TF) regulatory networks. Enrichment was performed using ChEA 2022 (ChIP-X Enrichment Analysis) and Rummagene Transcription Factor Co-regulation libraries within Enrichr. Significance was assessed using hypergeometric testing with Benjamini–Hochberg FDR correction; TFs with adj. *p* < 0.05 were considered significant, and those with adj. *p* < 0.1 were retained as suggestive.

## 3. Results

### 3.1. Polyp Development in CC-F1 Mice

Previous studies have demonstrated variation in susceptibility to intestinal polyp development across classical inbred mouse strains, leading to the categorization of polyp-resistant and polyp-susceptible strains [22]. Collaborative Cross (CC) mice, which contain unique combinations of homozygous chromosome segments from eight founder strains (A/J, B6J, 129S1/SvImJ, NOD/ShiLtJ, NZO/HILtJ, CAST/EiJ, PWK/PhJ, and WSB/EiJ), offer an opportunity to explore more extreme phenotypes than those observed in the founder strains [18].

In this study, we evaluated intestinal polyp development across 14 F1-CC strains. There was significant variation in polyp counts, with certain strains exhibiting markedly higher susceptibility to polyp formation compared to more resistant strains. This variability extended beyond the traditionally classified resistant and susceptible strains. Using a cohort of 260 mice from 14 CC-C57BL/6 J-*Smad4*^tm1Mak^ F1 lines, we mapped quantitative trait locus (QTL) modifiers of Smad4 based on polyp counts in both the small intestine and colon [17].

**Figure 1:**
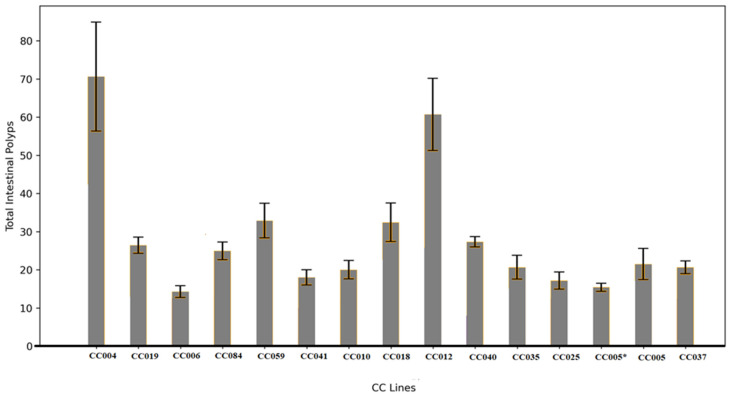
Total intestinal polyp count (±SE) among CC-F1 crosses carrying the *Smad4*+/− mutation at 80 weeks of age. The data represent phenotyping of 260 F1 hybrid mice derived from 14 Collaborative Cross (CC) lines. The Y-axis denotes the average number of polyps; the X-axis indicates the CC-Smad4+/− F1 hybrid lines. Statistical analysis was performed using one-way ANOVA; *p* < 0.05.

A one-way ANOVA revealed significant variation in total polyp counts among the different CC-*Smad4*^tm1Mak^ F1 lines. Polyp counts were approximately normally distributed, suggesting that both genetic and environmental factors contribute to this trait. To investigate whether different segments of the intestine exhibited distinct patterns of polyp distribution, we subdivided the small intestine into three sections: proximal (SB1), middle (SB2), and distal (SB3), along with the colon. Polyps in each segment were categorized based on size and analyzed independently. Analysis revealed significant inter-line differences in polyp burden across the SB1 region of the small intestine (Figure 1), with polyp counts presented as mean ± standard error (SE), supporting the presence of underlying genetic modifiers in the Collaborative Cross background.

### 3.2. QTL Analysis

To identify genetic loci associated with susceptibility to intestinal polyp development, we conducted genome-wide QTL mapping using the R/qtl2 framework. Polyp counts were measured across different regions of the gastrointestinal tract, including the proximal (SB1), middle (SB2), and distal (SB3) segments of the small intestine, as well as the colon. Analyses were performed on the full F1 cohort as well as stratified by sex, and QTL were categorized based on permutation-derived genome-wide significance thresholds, including significant, suggestive, and exploratory loci as defined by the empirical LOD distributions.

**Figure 2:**
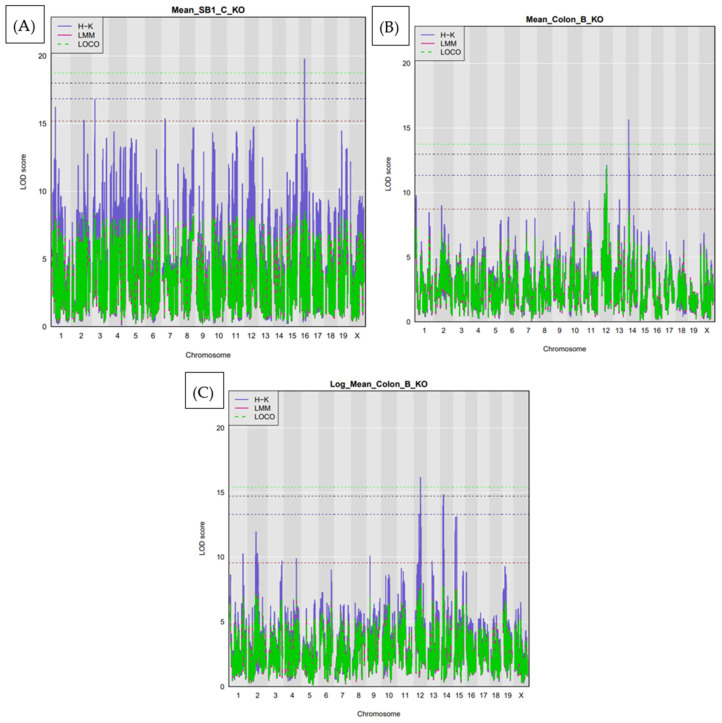
QTL mapping for polyp susceptibility at the genome-wide significance threshold (permutation-derived). (**A**) Significant QTL identified on chromosome 16 (designated *Ipsl*1) with a peak at 26.06 Mb, associated with polyp burden in the proximal small intestine (SB1) of F1-*Smad4* knockout mice. The 95% confidence interval spans 0.82 Mb, suggesting a highly localized genetic effect. (**B**) A second QTL, *Ipsl*2, was mapped to chromosome 14 with a peak at 8.2 Mb, linked to colon polyp development. The corresponding confidence interval covers 8.79 Mb, indicating a broader region of interest. (**C**) An additional colon-associated QTL, *Ipsl*3, was detected on chromosome 12 with a peak at 33.16 Mb and a confidence interval of 0.27 Mb, suggesting a discrete, independently acting genetic contributor to colon polyp susceptibility.

At the genome-wide significance threshold determined by permutation testing, ten significant QTL were detected, as detailed in Table 1. These QTL were designated Ipsl1 to Ipsl10 for Intestinal Polyposis Susceptibility locus (Ipsl). In the full cohort, three loci reached this threshold. A QTL on chromosome 16, designated IPSL1, was associated with polyp counts in the SB1 region and mapped to a narrow interval spanning 26.05–26.06 Mb. In the colon, two additional QTL were identified: IPSL2 on chromosome 14, peaking at 8.2 Mb with a confidence interval of 8.16–8.22 Mb, and IPSL3 on chromosome 12, linked to log-transformed polyp counts, spanning 32.93–33.17 Mb. These findings suggest that multiple genomic regions modulate polyp burden across different intestinal compartments. (Figure 2A–C).

**Figure 3:**
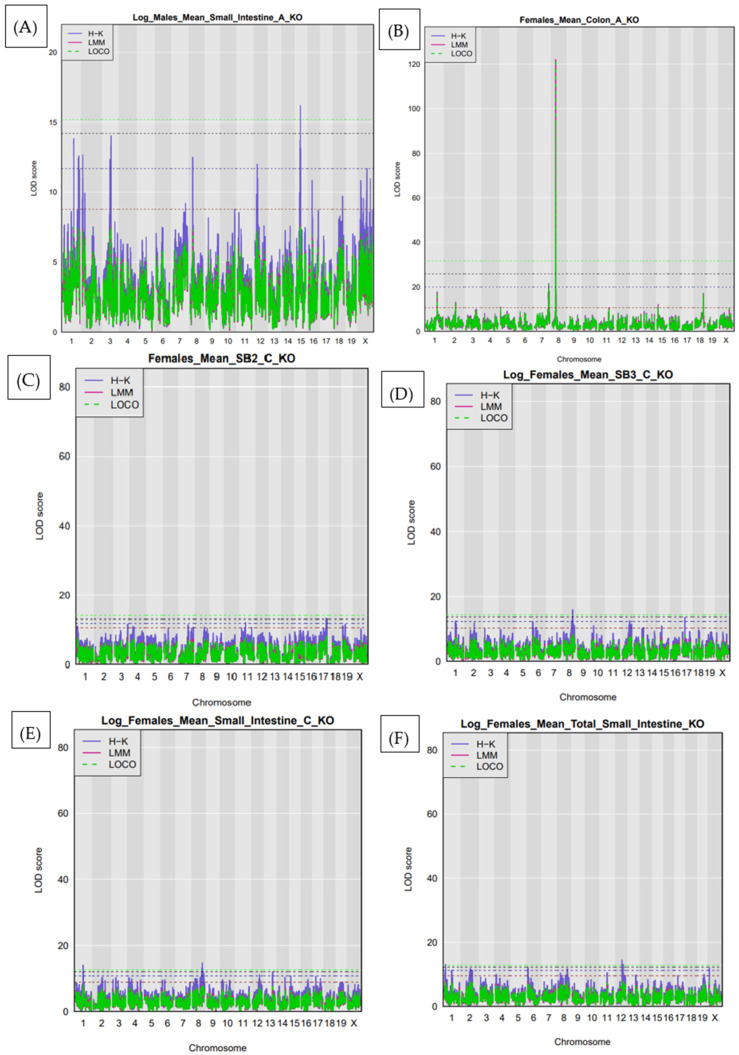
Sex-stratified QTL mapping at the genome-wide significance threshold. (**A**) Significant QTL (*Ipsl*4) detected in male mice, mapped to chromosome 15 at 25.83 Mb, associated with polyp burden in the small intestine (SB2 region). (**B**–**F**) QTL identified in female mice, revealing multiple loci associated with polyp susceptibility across intestinal regions. (**B**) *Ipsl*5 on chromosome 8 (60.26 Mb) for the small intestine (SB3). (**C**) *Ipsl*6 on chromosome 8 (19.45 Mb) for colon polyps. (**D**) *Ipsl*7 on chromosome 8 (58.75 Mb) for SB3; (**E**) *Ipsl*8 and *Ips*l9 on chromosomes 1 and 8, respectively, for the small intestine (SB3). (**F**) *Ipsl*10 on chromosome 12 (37.78 Mb) for total small intestine polyp counts.

In the male-only analysis, one significant QTL was identified. This locus, *Ipsl*4, mapped to chromosome 15 at 25.83 Mb and was associated with polyp counts in the SB2 region. The confidence interval for this locus was 25.71–25.93 Mb, indicating a highly localized effect (Figure 3A). In contrast, the female-only analysis revealed six significant QTL. Three of these were found on chromosome 8, including *Ipsl*5 (60.26 Mb), *Ipsl*6 (19.45 Mb), and *Ipsl*7 (58.75 Mb), all associated with either SB3 or colon polyp traits. Additional loci included IPSL8 on chromosome 1 at 46.65 Mb, *Ipsl*9 also on chromosome 8 at 60.16 Mb, and *Ipsl*10 on chromosome 12 at 37.78 Mb, which were associated with total small intestine polyp counts. These results highlight both common and sex-specific genetic influences on intestinal tumorigenesis, as shown in Figure 3B–F.

At the suggestive threshold defined by permutation analysis, ten additional QTL were identified (Table 2). In the full cohort, one QTL—*Ipsl1*1—was detected on chromosome 14 at 12.25 Mb, associated with log-transformed colon polyp counts and spanning a 4.09 Mb interval. In males, four loci emerged: *Ipsl*12, a broad QTL on chromosome 1 covering over 76 Mb; *Ipsl*13 on chromosome 3 at 38.78 Mb with no span; *Ipsl*14 on chromosome 1 at 7.56 Mb; and *Ipsl*15 on chromosome 15 at 21.39 Mb. The relatively wide confidence intervals for some of these loci suggest the possibility of complex or polygenic influences in male mice.

In females, five QTL were detected at the suggestive threshold defined by permutation analysis. *Ipsl*16, located on chromosome 17 at 46.37 Mb, was associated with SB2 polyp burden and exhibited a broad interval extending to 52.97 Mb. *Ipsl*17 and *Ipsl*18 were also located on chromosome 17, with overlapping positions and smaller confidence intervals. *Ipsl*19 was a point-wise QTL on the same chromosome at 17.37 Mb, and *Ipsl*20, on chromosome 1 at 8.79 Mb, spanned a narrow interval of 8.75–8.86 Mb. These female-specific loci may point to regulatory regions affecting intestinal phenotypes in a sex-dependent manner.

At a more permissive exploratory threshold below the suggestive level, numerous exploration QTL were detected across all groups and intestinal regions. These loci, presented in Supplementary Tables S1–S3, encompass a broader genetic architecture and may reflect the cumulative contributions of moderate-effect variants. While these loci fall below conventional significance thresholds, they serve as hypothesis-generating candidates for future studies, including fine-mapping, eQTL analysis, and functional validation.

### 3.3. Founder Strain Effects at Significant QTL

**Figure 4:**
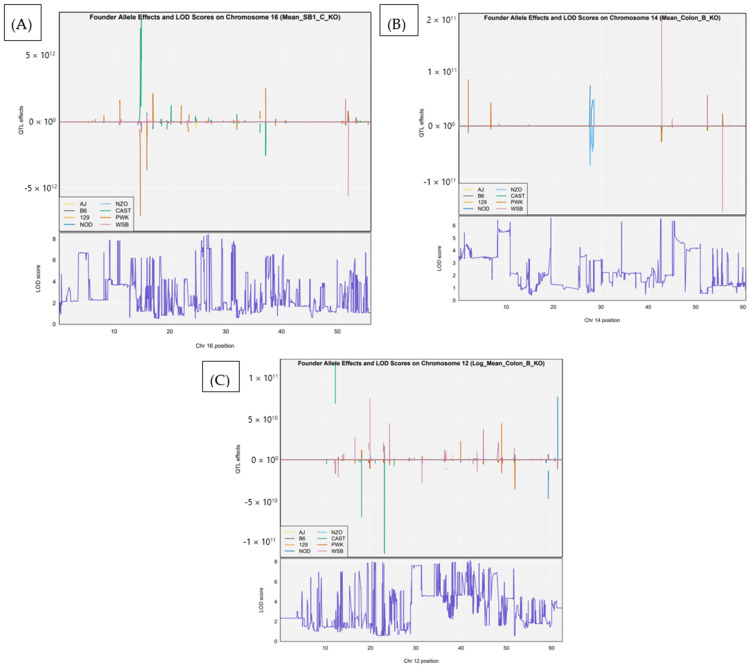
Founder strain effects at QTL peaks for polyp susceptibility. Founder allele effects and corresponding LOD scores are shown for the most significant QTL detected at the genome-wide significance threshold (Figure 2): (**A**) *Ipsl*1 on chromosome 16, associated with polyp burden in the SB1 region. (**B**) *Ipsl*2 on chromosome 14 is associated with colon polyp counts. (**C**) *Ipsl*3 on chromosome 12, associated with log-transformed colon polyp counts. The top panels display estimated founder allele effects at each locus, with color-coded lines representing the eight CC founder strains (AJ, B6, 129, NOD, NZO, CAST, PWK, and WSB). The bottom panels show LOD score profiles across each chromosome. These results highlight strain-specific contributions to polyp susceptibility, with notable peaks in PWK, CAST, and WSB alleles across different loci.

To assess the genetic contributions of individual CC founder strains to polyp susceptibility, we examined allele-specific effects at the peak loci of the most significant QTL (*Ipsl*1–*Ipsl*3). Founder effect plots revealed distinct strain-specific contributions across loci (Figure 4A–C). At *Ips*l1 (chromosome 16), associated with SB1 polyp counts, the PWK and CAST alleles displayed the largest effects, suggesting a major influence on proximal small intestine susceptibility. At *Ipsl*2 (chromosome 14), which was linked to colon polyp burden, prominent effects were observed from the NZO and WSB founder alleles. For *Ipsl*3 (chromosome 12), associated with log-transformed colon polyp counts, the CAST, PWK, and WSB strains again showed strong deviation from the baseline, indicating consistent contributions to polyp modulation across multiple intestinal regions. These findings underscore the heterogeneous genetic architecture underlying polyp susceptibility and highlight specific founders with pleiotropic or region-specific effects. Founder strain effect plots for the three major QTL (*Ipsl*1–*Ipsl*3) revealed distinct allele-specific contributions, particularly from PWK, CAST, and WSB founders, supporting their role in modulating intestinal polyp susceptibility (Figure 4).

**Figure 5:**
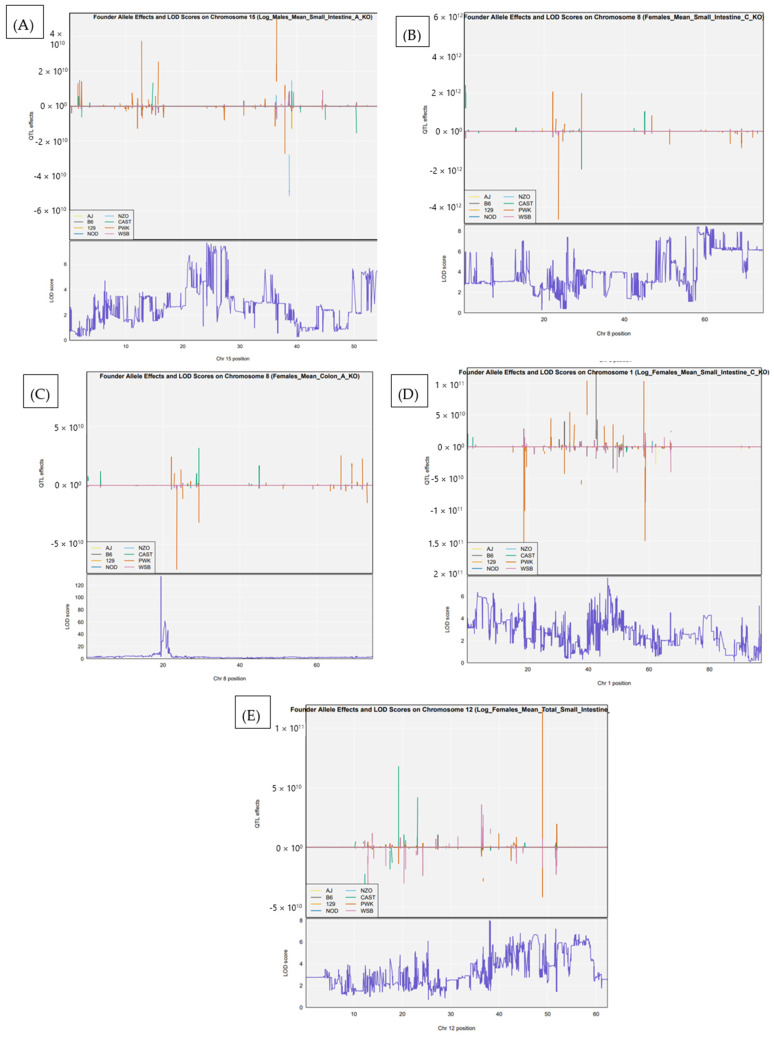
Founder allele effects and LOD profiles at sex-stratified QTL for intestinal polyp traits (**A**) Male-specific QTL (*Ipsl*4) on chromosome 15, associated with small intestine polyp counts in the SB2 region (trait: Log_Males_Mean_Small_Intestine_A_KO). (**B**) Female-specific QTL (*Ipsl*5) on chromosome 8, associated with small intestine polyp burden (trait: Females_Mean_Small_Intestine_C_KO). (**C**) Female-specific QTL (*Ips*l6) on chromosome 8, associated with colon polyp burden (trait: Females_Mean_Colon_A_KO). (**D**) Female-specific QTL (*Ipsl*7) on chromosome 1, associated with SB3 polyp counts (trait: Log_Females_Mean_SB3_C_KO). (**E**) Female-specific QTL (*Ipsl*8) on chromosome 12, associated with total small intestine polyps (trait: Log_Females_Mean_Total_Small_Intestine_KO).

To identify sex-specific genetic effects on intestinal polyp development, we performed separate QTL mapping analyses in male and female cohorts. This analysis revealed six additional significant loci that reached the genome-wide significance threshold in either sex. In males, one QTL (*Ipsl*4) on chromosome 15 (Figure 5A) was significantly associated with polyp counts in the small intestine (SB2 region), with CAST and NOD alleles contributing the strongest effects at the peak. In females, five distinct QTL were detected across multiple intestinal compartments. A locus on chromosome 8 (IPSL5) was associated with polyp counts in the small intestine (Figure 5B), while a second signal on the same chromosome (IPSL6) was linked to polyp burden in the colon (Figure 5C). Additional female-specific QTL included IPSL7 on chromosome 1 for SB3 counts (Figure 5D), IPSL8 on chromosome 12 for total small intestinal polyps (Figure 5E), and IPSL9 on chromosome 15, overlapping but distinct from the male-specific locus. These findings underscore the sexually dimorphic architecture of polyp susceptibility, with chromosome 8 emerging as a shared regulatory locus for both small intestine and colon phenotypes in females. Founder strain effect plots for loci reaching the suggestive threshold defined by permutation analysis are provided in Supplementary Materials.

### 3.4. Pathway Enrichment Analysis

Pathway enrichment results revealed several biologically plausible candidates, though most enrichments fell below strict statistical thresholds. In WikiPathways, the IL-2 and IL-7 signaling pathways were enriched via NMI and STAM2, implicating immune regulatory roles [32]. The proteasome degradation pathway included PSMD6, aligning with known functions in protein turnover and ubiquitin signaling [33]. Interestingly, CACNB4 appeared in the Dravet syndrome pathway (WP5298), consistent with its role in calcium channel regulation in neuronal contexts [34], while RPRM contributed to p53 signaling, associated with DNA damage response [35].

Analysis using CORUM identified several high-confidence protein complexes, including the IFP35–NMI and Emerin–Actin–NMI complexes, suggesting involvement of NMI in chromatin architecture and transcriptional control [36]. STAM2 appeared in multiple complexes related to endosomal sorting and EGFR trafficking, supporting its role in signal transduction and vesicular trafficking [37].

Reactome pathway enrichment reinforced several of these findings. The Ub-specific processing protease pathway included PSMD6, STAM2, and ATXN7, implicating protein degradation machinery. The nicotinamide salvaging pathway, represented by NAMPT, aligns with its regulatory role in NAD biosynthesis and SIRT1 activity [38]. Enrichment of the Rho GTPase cycle, involving RND3 and FMNL2, pointed to cytoskeletal dynamics and signaling functions [39]. While many Reactome enrichments had adjusted *p*-values > 0.1, their biological coherence strengthens the relevance of these genes in the QTL phenotypes examined.

Collectively, these results suggest that genes located within QTL may converge on pathways involved in transcriptional regulation, immune signaling, protein degradation, and neuronal excitability. Although direct expression data from intestinal tissues were not available in this study, the existing literature supports the involvement of several candidate genes in epithelial biology and tumorigenesis [40,41].

### 3.5. Gene Functional Analysis

Functional characterization of genes within the identified QTL regions was performed to provide additional context for the observed phenotype. Several genes were found to participate in enriched pathways and protein complexes, suggesting potential roles in relevant biological processes. However, most pathway enrichments were suggestive and did not reach conventional statistical significance thresholds (FDR < 0.05).

To summarize these associations, Table 3 presents each gene alongside its most relevant enriched pathway or protein complex, including the source database and adjusted *p*-values. These results provide a framework for interpreting the potential biological functions of candidate genes identified within the QTL regions.

One of the most prominent candidates, PSMD6, was consistently enriched across Reactome and CORUM, indicating its integral role in the ubiquitin–proteasome system. This gene encodes a non-ATPase subunit of the 26S proteasome, essential for protein degradation and turnover [33]. Its recurrence in pathways such as “Ub-specific processing proteases” and “proteasome-mediated degradation” implies a mechanistic link between proteostasis and the trait captured by the QTL.

Another recurrent gene, STAM2, was found in multiple protein complexes involved in endosomal sorting and receptor signaling, including the RIN1–STAM2–EGFR complex and the ESCRT machinery [37]. These complexes are critical for trafficking membrane-bound receptors, including EGFR, and thereby influence signal transduction pathways. STAM2 also featured in immune-related pathways, including IL-2 and IL-7 signaling, highlighting a potential interface between vesicle trafficking and cytokine signaling.

NAMPT was enriched in the nicotinamide salvaging pathway, underscoring its role in NAD+ biosynthesis and cellular metabolism [38]. Its involvement in metabolic regulation may reflect a broader metabolic component underlying the QTL phenotype. Similarly, NMI (N-myc and STAT interactor) was identified in multiple CORUM complexes and has known functions in transcriptional regulation, particularly in cytokine and interferon response pathways [36].

CACNB4, a beta subunit of voltage-dependent calcium channels, appeared in both the Dravet Syndrome pathway and neuronal system signaling cascades. This is consistent with its role in neuronal excitability and synaptic transmission and suggests that the QTL may capture variation in neurophysiological processes [34].

Genes like RND3 and FMNL2, involved in Rho GTPase signaling, were linked to pathways regulating cytoskeletal reorganization and cell migration. RND3 negatively regulates RhoA activity and modulates neurite outgrowth [39], while FMNL2 is a formin family member involved in Actin polymerization and Golgi organization [39]. These findings highlight the possible influence of cytoskeletal remodeling in the phenotype associated with QTL.

**Figure 6:**
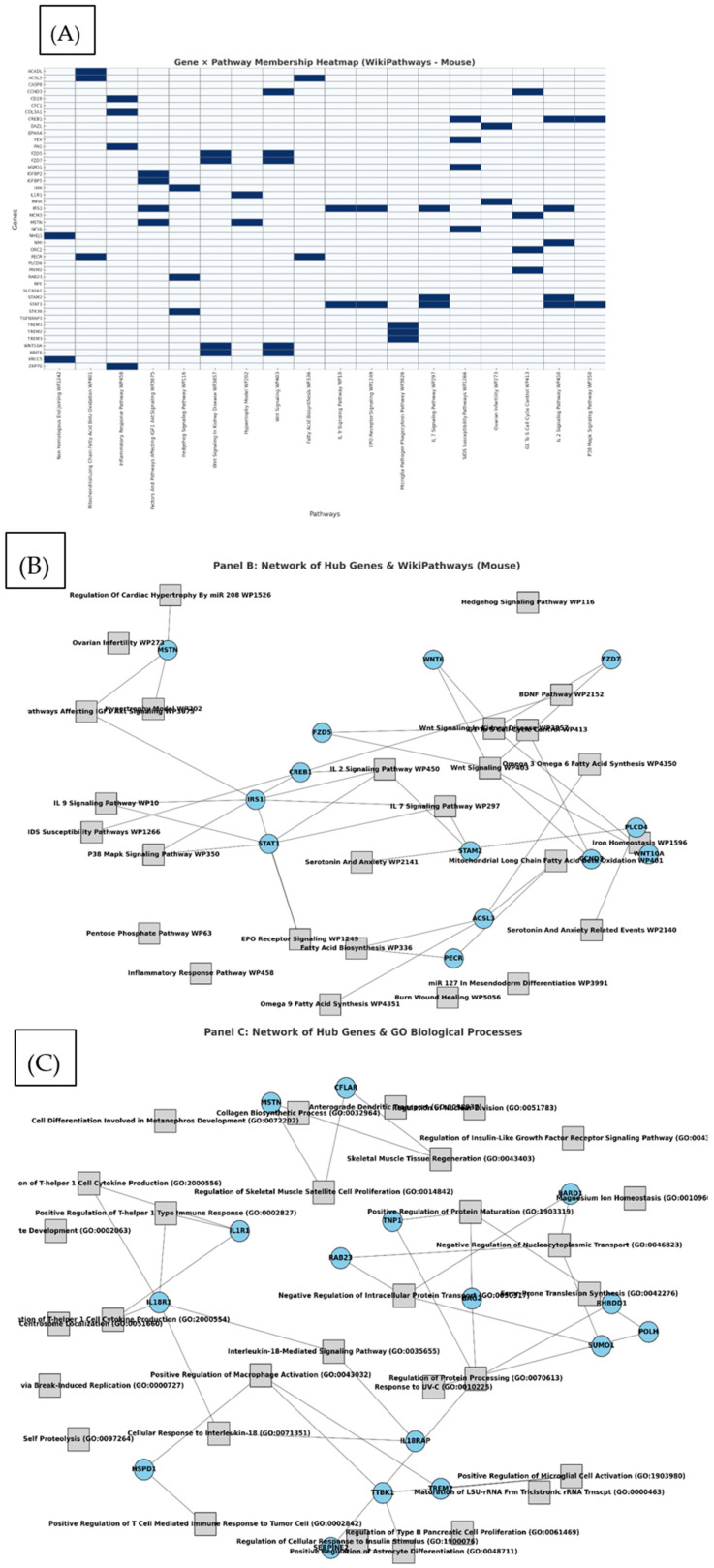
(**A**) Gene × pathway heatmap of QTL-associated genes (rows) and the top enriched pathways (columns) identified from WikiPathways (Mouse). Blue shading indicates gene membership in a pathway (1 = present). Only pathways with at least two associated genes are shown. (**B**) Network representation of QTL-associated genes and their functionally enriched pathways. This network depicts the top 40 enriched pathways identified from QTL-associated genes, and the hub genes that participate in two or more of these pathways. Edges connect genes to pathways in which they were annotated. Labels are shown for both genes and pathways to emphasize the connectivity structure. This representation highlights the convergence of multiple candidate genes onto shared functional pathways, suggesting pleiotropic mechanisms underlying QTL-associated traits. (**C**) Similar network using the top GO Biological Processes. Edges link hub genes to enriched biological terms, revealing convergence on shared cellular functions.

A network visualization of the QTL-associated genes and their enriched functional pathways is presented in Figure 6, highlighting key regulators such as *STAM2*, *PSMD6*, and *NAMPT*, which contribute to multiple biological processes.

Together, the convergence of these genes across signaling, metabolism, protein degradation, and neuronal pathways supports a multifaceted genetic architecture underlying the identified QTL. The functional diversity of these genes emphasizes the importance of integrating pathway and complex-level annotations to decode complex trait loci.

### 3.6. Gene–Pathway Networks and Hub Gene Architecture

To better understand the functional convergence of QTL-associated genes, we constructed gene–pathway networks based on the top 30 enriched pathways from WikiPathways (2024, Mouse) and GO Biological Processes (2025). In both datasets, several genes appeared in multiple pathways, forming a set of hub genes likely to play pleiotropic roles across biological processes.

In the WikiPathways-derived network, hub genes such as *STAM2*, *PSMD6*, and *NAMPT* connected to pathways related to immune signaling, protein degradation, and NAD+ metabolism, respectively (Figure 6B). The GO network revealed similar patterns, with shared genes linking vesicle trafficking, ubiquitin signaling, and developmental processes (Figure 6C). These results support the hypothesis that QTL intervals contain regulators with broad biological influence.

To validate and contextualize these findings, we also generated a binary gene–pathway heatmap of the enriched WikiPathways. This revealed subsets of genes that cluster in distinct biological modules (Figure 6A), reinforcing the functional overlap observed in the network analysis.

### 3.7. Transcription Factor Associations

Additionally, transcription factor enrichment yielded several significant results. Using ChEA 2022 and the Rummagene transcription factor co-regulation library, we identified multiple transcription factors significantly enriched at FDR < 0.05, with additional regulators emerging at FDR < 0.1. These findings indicate that genes mapping to the identified QTL may be under coordinated transcriptional control. A complete list of enriched transcription factors and their adjusted *p*-values is provided in Supplementary Table S4.

## 4. Discussion

This study leverages the genetic diversity of Collaborative Cross (CC) mice to investigate modifier loci influencing polyp development in *Smad4*-deficient mice, a model for Juvenile Polyposis Syndrome (JPS). The substantial phenotypic variation observed across F1 offspring reflects the complex, multigenic nature of JPS, consistent with previous findings that patient outcomes vary despite identical germline mutations [42,43].

The identification of significant QTL on chromosomes 2, 12, 14, and 16 suggests the existence of multiple genetic loci that modulate intestinal polyp susceptibility. The loci detected, particularly *IPSL1* on chromosome 16 and *Ipsl2/Ipsl3* on chromosomes 14 and 12, corroborate earlier studies that have emphasized the importance of background genetics in tumor susceptibility [18,26]. The strong effect observed from the PWK/PhJ founder haplotype highlights how wild-derived alleles may contribute disproportionately to phenotype variability, a trend also noted in CC-based studies of Apc^Min^ tumor models [22].

Several candidate genes were identified within the QTL regions, including *PSMD6*, *STAM2*, and *NAMPT*, which have been implicated in cancer-related processes, supporting their potential translational relevance. For example, *PSMD6*, a component of the 26S proteasome, has been reported to be upregulated in colon cancer and is associated with tumor progression, highlighting a role for proteasomal activity in maintaining oncogenic signaling [44]. Similarly, *NAMPT*, a key enzyme in NAD+ biosynthesis, has been extensively linked to colorectal cancer, where it promotes tumor growth, metabolic reprogramming, and cancer stem cell maintenance [41,45]. In addition, *NAMPT* has been shown to regulate key pathways such as SIRT1 signaling and cellular metabolism, both of which are critical for tumor progression and therapy resistance [40]. Although *STAM2* has been less directly studied in colorectal cancer, its role in endosomal trafficking and receptor signaling suggests potential involvement in pathways such as EGFR signaling, which are central to intestinal epithelial biology [46]. These findings support the biological plausibility of the identified loci and highlight potential mechanisms through which genetic background may influence polyp susceptibility in Smad4-deficient mice. However, direct validation in human JPS cohorts will be required to confirm their role as genetic modifiers.

Pathway enrichment of genes within these QTL pointed to biological systems already implicated in colorectal cancer and JPS pathogenesis, including TGF-β and Wnt signaling, proteasomal degradation, and cytokine-mediated immune regulation [10,47,48]. For example, *PSMD6*, identified in both Reactome and CORUM pathways, participates in proteasomal degradation and may influence tumor suppressor protein turnover, consistent with its proposed role in cancer biology [49].

Other recurrent genes, such as *STAM2* and *NMI* were involved in immune signaling (e.g., IL-2, IL-7) and transcriptional regulation, respectively, linking inflammation and immune surveillance to polyp burden—an increasingly recognized axis in tumorigenesis [32,36]. Additionally, genes such as *CACNB4* and *RND3*, more commonly associated with neuronal or cytoskeletal function, may reflect the broader cellular architecture and signaling dynamics that influence epithelial organization and tumor progression [34,50].

These findings align with the observed phenotypic variability in JPS. They suggest that differential expression or mutation of these genes may contribute to variability even among individuals carrying identical Smad4 mutations [4]. By elucidating candidate modifier loci and pathways, this study adds to the growing understanding that JPS is influenced by more than just a single causative mutation.

Importantly, the loci identified in this study differ from those reported in ApcMin-based models, suggesting that modifier effects are influenced by the underlying tumorigenic pathway and highlighting a context-specific genetic architecture.

A limitation of this work is the absence of detailed histopathological and protein-level validation of the identified phenotypes. While the primary aim was genetic mapping, future studies incorporating immunohistochemistry and molecular profiling will be important to further assess the functional impact of candidate modifier genes. In addition, the interpretation of sex-stratified QTL analyses requires some caution. Although the cohort included a relatively balanced number of males (n = 129) and females (n = 131), stratification reduces the effective sample size compared to the combined analysis. Consequently, some of the observed sex-specific differences may reflect reduced power to detect modest-effect loci rather than true biological dimorphism.

Together, these findings provide a framework for understanding how genetic background influences polyp susceptibility in *Smad4*-deficient models and highlight candidate pathways for future functional and translational studies.

## 5. Conclusions

This study identified novel genetic loci that modulate polyp development in a *Smad4*-deficient mouse model of Juvenile Polyposis Syndrome using the genetically diverse Collaborative Cross population. QTL mapping revealed multiple loci across several chromosomes, implicating genes involved in proteasome function, immune signaling, and cytoskeletal organization.

These findings provide insight into the polygenic nature of JPS and suggest that individuals with the same Smad4 mutation may experience differing clinical outcomes due to the influence of background genetic modifiers. The identification of genes such as *PSMD*6, *STAM*2, *NAMPT,* and *CACNB*4 supports further exploration of pathways like protein degradation and cytokine signaling in JPS pathophysiology.

By integrating genetic mapping with pathway and gene function analysis, our work lays the foundation for future translational studies aimed at improving risk stratification and informing surveillance strategies based on individual genetic backgrounds [17]. Validating these loci in human cohorts will be a crucial next step toward understanding the molecular underpinnings of phenotypic heterogeneity in hereditary gastrointestinal cancer syndromes.

## Figures and Tables

**Table 1:** QTL detected at genome-wide significance threshold for polyp development in the SB1 and colon regions. Summary of QTL identified on chromosomes 16, 14, and 12 for susceptibility to polyp formation in the SB1 and colon regions of F1-Smad4 knockout mice.

	Trait Name	Chromosome	QTL Name	Peak (Mb)	95% CI (Mb)	Size (Mb)
Full Cohort	Mean_SB1_C_KO	16	*Ipsl*1	26.06	26.05–26.06	0.82
	Mean_Colon_B_KO	14	*Ipsl*2	8.2	8.16–8.22	8.79
	log_Mean_Colon_B_KO	12	*Ipsl*3	33.16	32.93–33.17	0.27
Male population	Log_Males_Mean_Small_Intestine_A_KO	15	*Ipsl*4	25.83	25.71–25.93	0.23
Female population	Females_Mean_Small_Intestine_C_KO	8	*Ipsl*5	60.26	58.2–60.3	2.1
	Females_Mean_Colon_A_KO	8	*Ipsl*6	19.45	19.45–19.45	0.0
	Log_Females_Mean_SB3_C_KO	8	*Ipsl7*	58.75	58.71–59.82	1.11
	Log_Females_Mean_Small_Intestine_C_KO	1	*Ipsl*8	46.65	46.52–46.97	0.44
	Log_Females_Mean_Small_Intestine_C_KO	8	*Ipsl*9	60.16	60.14–60.24	0.1
	Log_Females_Mean_Total_Small_Intestine_KO	12	*Ipsl*10	37.78	37.77–37.79	0.01

**Table 2:** QTL detected at suggestive significance threshold for polyp development in the SB3 and colon regions. Listing of QTL detected on chromosomes 2 and 14 for polyp development in the distal small intestine (SB3) and colon regions, along with their peak locations and confidence intervals.

	**Trait Name**	**Chromosome**	**QTL Name**	**Peak (Mb)**	**95% CI (Mb)**	**Size (Mb)**
Full Cohort	Log_Mean_Colon_B_KO	14	*Ipsl1*1	12.25	8.16–12.25	4.09
Males’ population	Males_Mean_SB1_A_KO	1	*Ipsl*12	6.89	6.82–83.25	76.43
	Males_Mean_Small_Intestine_A_KO	3	*Ipsl*13	38.78	38.78–38.78	0.0
	Log_Males_Mean_SB1_A_KO	1	*Ipsl*14	7.56	6.88–7.58	0.7
	Log_Males_Mean_SB1_A_KO	15	*Ipsl*15	21.39	20.73–21.62	0.89
Females’ population	Females_Mean_SB2_C_KO	17	*Ipsl*16	46.37	46.35–52.97	6.62
	Females_Mean_Small_Intestine_C_KO	1	*Ipsl*17	46.35	46.35–46.35	0.0
	Log_Females_Mean_SB2_C_KO	17	*Ipsl*18	46.37	46.35–46.45	0.09
	Log_Females_Mean_SB3_C_KO	17	*Ipsl*19	17.37	17.37–17.37	0.0
	Log_Females_Mean_Total_Small_Intestine_KO	1	*Ipsl*20	8.79	8.75–8.86	0.11

**Table 3:** Summary of pathway and protein complex enrichment for genes located within QTL regions. Each gene is linked to representative enriched pathways or complexes, along with the source database (e.g., Reactome, CORUM, WikiPathways), adjusted *p*-values, and associated functional roles.

Gene	Pathway/Complex	Source	Adjusted *p*-Value	Functional Role
PSMD6	Ub-specific Processing Proteases	Reactome	0.10	Proteasomal degradation
STAM2	RIN1–STAM2–EGFR Complex	CORUM	0.059	Endosomal sorting, receptor signaling
NAMPT	Nicotinamide Salvaging	Reactome	0.11	NAD+ biosynthesis
NMI	IFP35–NMI Complex	CORUM	0.059	Transcription regulation, immune response
CACNB4	Dravet Syndrome Pathway	WikiPathways	0.34	Neuronal signaling, calcium channels
RND3	Rho GTPase Cycle	Reactome	0.21	Cytoskeletal regulation, neurite outgrowth
FMNL2	Rho GTPase Effectors	Reactome	0.82	Actin dynamics, Golgi organization

## Data Availability

The datasets generated and analyzed during the current study are available at https://doi.org/10.6084/m9.figshare.31986828 (accessed on 25 April 2026).

## Supplementary Materials

The following supporting information can be downloaded at https://www.mdpi.com/article/10.3390/cells15100853/s1. Figure S1: QTL mapping results—full cohort; Figure S2: Founder allele effects—full cohort; Figure S3: QTL mapping results—male subset; Figure S4: Founder allele effects—male subset; Figure S5: QTL mapping results—female subset; Figure S6: Founder allele effects—female subset; Table S1: QTL detected at exploratory threshold for polyp development across different intestinal segments (whole population); Table S2: QTL detected at exploratory threshold for polyp development across different intestinal segments (male population); Table S3: QTL detected at exploratory threshold for polyp development across different intestinal segments (female population); Table S4: Significant transcription factor enrichment analysis using the ChEA 2022 database. Table S5: Significant transcription factor enrichments from Rummagene.

## Abbreviations

The following abbreviations are used in this manuscript:

JPS	Juvenile Polyposis Syndrome
QTL	Quantitative Trait Locus or Loci
CC	Collaborative Cross
CRC	Colorectal Cancer
SB1	Proximal Small Intestine
SB2	Middle Small Intestine
SB3	Distal Small Intestine
IPSL	Intestinal Polyposis Susceptibility Locus
KO	Knockout
FDR	False Discovery Rate
GO	Gene Ontology
