# Supplementary material for: Mapping Genetic Modifiers of Polyp Formation in Smad4-Deficient Juvenile Polyposis Using the Collaborative Cross Mouse Population
Source: Cells. 2026 May 7;15(10):853. doi: 10.3390/cells15100853 (PMC13204083; doi:10.3390/cells15100853)
Supplement: Supplementary file 1 [file cells-15-00853-s001.zip › cells-4227154-supplementary.pdf]

Supplementary Table S1: QTL Detected at 0.5 LOD Threshold for Polyp Development across Different Intestinal Segments (whole population)

Detailed presentation of QTL across chromosomes 1, 2, 3, 7, and 15 for polyp development in the small intestine and colon, with corresponding confidence intervals.

lodcolumn	chr	Peak (Mb)	95% CI (Mb)	Size (Mb)
Mean_SB1_A_KO	1	67.04	67.03–82.58	15.55
Mean_SB1_A_KO	6	3.31	0.5–3.79	3.29
Mean_SB1_A_KO	7	83.91	45.26–83.98	38.71
Mean_SB1_A_KO	9	72.14	71.13–72.73	1.59
Mean_SB1_A_KO	11	9.74	9.38–9.85	0.47
Mean_SB1_A_KO	14	14.82	14.82–14.82	0.0
Mean_SB1_A_KO	15	55.78	55.77–55.81	0.03
Mean_SB1_C_KO	1	16.88	16.8–16.89	0.09
Mean_SB1_C_KO	2	66.36	65.97–66.4	0.43
Mean_SB1_C_KO	3	11.41	11.3–11.58	0.28
Mean_SB1_C_KO	7	13.51	13.5–15.26	1.76
Mean_SB1_C_KO	15	57.16	55.27–57.4	2.14
Mean_SB1_KO	2	65.97	65.97–66.4	0.43

Mean_SB1_KO	3	11.41	11.3–11.58	0.28
Mean_SB1_KO	11	46.34	46.33–46.36	0.02
Mean_SB2_A_KO	18	51.7	51.64–51.78	0.14
Mean_SB2_B_KO	7	38.34	37.96–38.39	0.42
Mean_SB2_B_KO	10	7.93	7.93–7.93	0.0
Mean_SB2_B_KO	11	5.41	5.2–5.81	0.6
Mean_SB2_B_KO	18	51.78	51.77–52.0	0.23
Mean_SB2_B_KO	X	67.19	67.19–67.19	0.0
Mean_SB2_C_KO	4	60.71	60.61–61.53	0.92
Mean_SB2_C_KO	10	3.43	3.43–3.49	0.06
Mean_SB2_C_KO	12	37.77	36.92–42.17	5.25
Mean_SB2_C_KO	19	18.74	18.71–18.74	0.04
Mean_SB2_KO	4	60.54	60.52–61.26	0.74
Mean_SB2_KO	12	37.77	37.76–42.17	4.41
Mean_SB3_KO	2	51.48	50.39–54.34	3.95
Mean_SB3_KO	3	32.35	32.35–32.92	0.57
Mean_SB3_KO	8	59.75	58.71–59.75	1.05

Mean_Small_Intestine_C_KO	1	16.88	16.8–16.89	0.09
Mean_Small_Intestine_C_KO	4	60.74	8.67–61.79	53.12
Mean_Small_Intestine_C_KO	7	86.74	13.5–86.75	73.25
Mean_Small_Intestine_C_KO	8	63.81	63.77–63.84	0.07
Mean_Small_Intestine_C_KO	11	47.54	46.05–47.57	1.52
Mean_Small_Intestine_C_KO	12	43.97	41.94–43.97	2.03
Mean_Small_Intestine_C_KO	16	26.06	26.05–26.64	0.59
Mean_Small_Intestine_C_KO	19	18.74	18.71–18.74	0.04
Mean_Total_Small_Intestine_KO	4	8.68	2.6–77.5	74.89
Mean_Total_Small_Intestine_KO	7	86.74	13.43–86.75	73.33
Mean_Total_Small_Intestine_KO	11	46.07	46.05–47.55	1.5
Mean_Total_Small_Intestine_KO	12	43.97	41.94–43.97	2.03
Mean_Total_Small_Intestine_KO	16	26.06	26.05–26.64	0.59
Mean_Colon_B_KO	1	0.65	0.19–1.87	1.68
Mean_Colon_B_KO	2	39.24	39.24–39.32	0.08
Mean_Colon_B_KO	10	34.74	34.29–35.56	1.27

Mean_Colon_B_KO	11	32.22	21.48–33.02	11.55
Mean_Colon_B_KO	12	35.39	32.32–35.4	3.08
Mean_Colon_B_KO	13	33.98	33.98–33.98	0.0
Mean_Colon_C_KO	3	11.41	11.3–11.58	0.28
Mean_Colon_C_KO	6	20.51	20.45–20.52	0.07
Mean_Colon_C_KO	12	49.73	46.9–49.73	2.83
Mean_Total_Colon_KO	3	11.41	11.3–11.58	0.28
Mean_Total_Colon_KO	6	20.51	20.45–20.52	0.07
Mean_Total_Colon_KO	12	48.34	47.67–49.73	2.06
Mean_Total_Intestinal_KO	2	65.97	65.97–65.97	0.0
Mean_Total_Intestinal_KO	9	16.13	16.12–16.18	0.06
Mean_Total_Intestinal_KO	12	45.28	38.52–45.28	6.76
Mean_Total_Intestinal_KO	19	45.5	45.34–45.64	0.3
Log_Mean_SB1_A_KO	1	67.04	8.75–67.08	58.32
Log_Mean_SB1_A_KO	6	0.75	0.5–0.79	0.29
Log_Mean_SB1_A_KO	7	45.26	45.26–45.28	0.02

Log_Mean_SB1_A_KO	11	9.74	9.66–10.2	0.54
Log_Mean_SB1_C_KO	4	3.87	3.83–3.88	0.05
Log_Mean_SB1_C_KO	7	15.25	15.22–15.26	0.04
Log_Mean_SB1_C_KO	8	14.21	14.21–31.49	17.27
Log_Mean_SB1_C_KO	16	26.05	25.86–26.52	0.66
Log_Mean_SB2_A_KO	11	9.85	9.74–10.14	0.4
Log_Mean_SB2_A_KO	18	51.7	51.64–51.72	0.08
Log_Mean_SB2_B_KO	1	68.52	68.52–70.56	2.04
Log_Mean_SB2_B_KO	10	7.93	7.93–7.93	0.0
Log_Mean_SB2_B_KO	14	42.7	42.47–42.74	0.27
Log_Mean_SB2_B_KO	X	67.19	67.19–67.65	0.46
Log_Mean_SB3_KO	1	6.17	6.02–8.86	2.84
Log_Mean_SB3_KO	2	50.39	50.39–54.34	3.95
Log_Mean_SB3_KO	6	0.79	0.74–0.85	0.1
Log_Mean_SB3_KO	12	51.67	51.61–51.74	0.13
Log_Mean_SB3_KO	19	35.5	31.6–45.64	14.04
Log_Mean_Small_Intestine_C_KO	1	18.75	18.72–18.78	0.06

Log_Mean_Small_Intestine_C_KO	7	86.25	86.24–86.26	0.02
Log_Mean_Small_Intestine_C_KO	10	3.43	3.37–3.43	0.06
Log_Mean_Small_Intestine_C_KO	12	37.78	37.77–37.79	0.01
Log_Mean_Small_Intestine_C_KO	13	49.1	49.1–49.1	0.0
Log_Mean_Total_Small_Intestine_KO	1	18.75	18.72–18.78	0.06
Log_Mean_Total_Small_Intestine_KO	4	8.68	2.6–77.4	74.8
Log_Mean_Total_Small_Intestine_KO	12	37.78	37.77–37.79	0.01
Log_Mean_Colon_B_KO	1	76.73	76.21–77.17	0.96
Log_Mean_Colon_B_KO	2	41.79	40.68–41.84	1.16
Log_Mean_Colon_B_KO	3	78.08	75.27–78.13	2.86
Log_Mean_Colon_B_KO	4	67.6	67.55–67.63	0.08
Log_Mean_Colon_B_KO	9	14.34	14.34–14.4	0.06
Log_Mean_Colon_B_KO	13	25.05	25.02–25.55	0.53
Log_Mean_Colon_B_KO	15	15.31	6.96–15.31	8.36
Log_Mean_Colon_C_KO	3	11.41	11.3–11.58	0.28
Log_Mean_Colon_C_KO	6	20.51	20.27–20.53	0.26
Log_Mean_Colon_C_KO	12	47.58	44.67–49.73	5.06

Log_Mean_Total_Colon_KO	3	11.41	11.3–11.58	0.28
Log_Mean_Total_Colon_KO	6	20.51	20.45–20.53	0.08
Log_Mean_Total_Colon_KO	12	49.72	44.67–49.73	5.06

Supplementary Table S2: QTL Detected at 0.5 LOD Threshold for Polyp Development across Different Intestinal Segments (male population)
Detailed presentation of QTL across chromosomes 1, 2, 3, 7, and 15 for polyp development in the small intestine and colon, with corresponding confidence intervals.

Table 6: LOD > 0.5 (Males)

lodcolumn	chr	Peak (Mb)	95% CI (Mb)	Size (Mb)
Males_Mean_SB1_A_KO	3	67.55	67.55–67.57	0.03
Males_Mean_SB1_A_KO	4	47.95	47.94–48.21	0.27
Males_Mean_SB1_A_KO	5	41.97	41.97–41.97	0.0
Males_Mean_SB1_A_KO	7	17.46	17.42–17.92	0.5
Males_Mean_SB1_A_KO	11	40.22	21.54–40.26	18.72
Males_Mean_SB1_A_KO	13	49.1	49.1–52.23	3.13
Males_Mean_SB1_A_KO	14	10.85	10.85–42.74	31.88
Males_Mean_SB1_A_KO	15	20.87	15.21–35.69	20.48
Males_Mean_SB1_A_KO	16	55.31	55.26–55.39	0.13
Males_Mean_SB1_A_KO	18	5.93	5.85–5.93	0.08
Males_Mean_SB1_A_KO	X	57.11	45.64–67.17	21.53
Males_Mean_SB1_C_KO	3	11.31	11.3–11.58	0.28

Males_Mean_SB1_C_KO	8	61.57	61.56–71.13	9.56
Males_Mean_SB1_C_KO	11	23.23	23.16–23.24	0.09
Males_Mean_SB1_C_KO	15	57.16	55.92–57.4	1.48
Males_Mean_SB1_C_KO	16	26.06	26.05–29.39	3.35
Males_Mean_SB1_C_KO	19	18.74	18.71–18.74	0.04
Males_Mean_SB1_KO	3	11.31	11.3–11.58	0.28
Males_Mean_SB1_KO	11	20.82	20.8–21.52	0.72
Males_Mean_SB1_KO	12	19.61	19.6–19.61	0.01
Males_Mean_SB1_KO	15	57.16	55.92–57.4	1.48
Males_Mean_SB2_B_KO	4	4.82	4.79–5.01	0.22
Males_Mean_SB2_B_KO	5	42.47	41.34–42.75	1.41
Males_Mean_SB2_B_KO	7	43.28	29.91–84.35	54.44
Males_Mean_SB2_B_KO	16	53.3	51.75–54.77	3.01
Males_Mean_SB3_B_KO	1	71.18	70.94–71.31	0.37
Males_Mean_SB3_B_KO	4	18.62	6.29–18.63	12.33
Males_Mean_SB3_B_KO	8	27.62	27.61–27.62	0.02

Males_Mean_SB3_B_KO	10	23.08	23.04–23.93	0.89
Males_Mean_SB3_B_KO	12	6.03	6.01–6.24	0.23
Males_Mean_SB3_B_KO	15	47.78	47.78–47.81	0.03
Males_Mean_SB3_B_KO	18	53.66	53.65–53.78	0.13
Males_Mean_Small_Intestine_A_KO	1	90.76	90.76–90.77	0.01
Males_Mean_Small_Intestine_A_KO	2	1.28	1.21–1.44	0.23
Males_Mean_Small_Intestine_A_KO	8	14.91	14.91–14.91	0.0
Males_Mean_Small_Intestine_A_KO	11	9.66	9.44–9.74	0.31
Males_Mean_Small_Intestine_A_KO	12	15.32	8.98–15.34	6.35
Males_Mean_Small_Intestine_A_KO	14	42.7	42.68–42.74	0.06
Males_Mean_Small_Intestine_A_KO	15	25.83	24.22–27.52	3.3
Males_Mean_Small_Intestine_A_KO	16	22.46	20.4–23.37	2.97
Males_Mean_Small_Intestine_A_KO	18	48.88	29.31–49.01	19.7
Males_Mean_Small_Intestine_A_KO	X	47.49	47.49–47.5	0.0
Males_Mean_Small_Intestine_C_KO	1	16.89	16.88–16.89	0.01
Males_Mean_Small_Intestine_C_KO	10	3.43	3.37–9.73	6.35

Males_Mean_Small_Intestine_C_KO	11	47.52	46.05–47.64	1.58
Males_Mean_Small_Intestine_C_KO	12	41.94	41.94–43.97	2.03
Males_Mean_Small_Intestine_C_KO	16	26.63	26.05–26.64	0.59
Males_Mean_Colon_A_KO	5	83.44	83.35–83.5	0.15
Males_Mean_Colon_A_KO	8	43.05	41.95–43.44	1.49
Males_Mean_Colon_A_KO	10	38.02	36.69–38.96	2.27
Males_Mean_Colon_B_KO	2	27.38	27.38–76.97	49.59
Males_Mean_Colon_B_KO	4	3.94	3.94–12.0	8.07
Males_Mean_Colon_B_KO	5	83.96	83.67–84.01	0.33
Males_Mean_Colon_B_KO	7	56.24	55.92–68.21	12.3
Males_Mean_Colon_B_KO	8	43.48	42.84–43.65	0.81
Males_Mean_Colon_B_KO	10	73.18	72.93–75.36	2.43
Males_Mean_Colon_B_KO	19	27.11	27.11–48.76	21.65
Males_Mean_Colon_B_KO	X	9.82	9.65–34.58	24.93
Log_Males_Mean_SB1_A_KO	3	80.8	67.55–81.17	13.62
Log_Males_Mean_SB1_A_KO	4	47.95	40.43–47.95	7.52

Log_Males_Mean_SB1_A_KO	5	12.72	12.7–40.58	27.88
Log_Males_Mean_SB1_A_KO	7	17.46	17.42–86.25	68.84
Log_Males_Mean_SB1_A_KO	9	67.3	56.3–67.35	11.05
Log_Males_Mean_SB1_A_KO	12	31.59	8.59–35.67	27.09
Log_Males_Mean_SB1_A_KO	14	10.85	10.85–10.86	0.0
Log_Males_Mean_SB1_A_KO	17	53.12	14.97–53.12	38.15
Log_Males_Mean_SB1_A_KO	19	46.93	46.89–47.1	0.2
Log_Males_Mean_SB1_A_KO	X	57.26	24.03–57.32	33.29
Log_Males_Mean_SB1_KO	3	38.8	38.8–38.92	0.12
Log_Males_Mean_SB1_KO	5	13.07	13.0–13.09	0.09
Log_Males_Mean_SB1_KO	7	17.32	14.98–17.34	2.36
Log_Males_Mean_SB1_KO	9	4.08	3.71–4.53	0.81
Log_Males_Mean_SB1_KO	11	20.86	20.74–21.55	0.81
Log_Males_Mean_SB1_KO	12	19.57	19.55–19.6	0.05
Log_Males_Mean_SB1_KO	14	40.76	40.72–40.9	0.18
Log_Males_Mean_SB1_KO	15	56.46	54.93–57.4	2.47

Log_Males_Mean_SB1_KO	16	9.25	9.25–9.25	0.0
Log_Males_Mean_SB1_KO	19	48.84	42.35–49.45	7.1
Log_Males_Mean_SB2_B_KO	1	8.7	8.67–8.77	0.1
Log_Males_Mean_SB2_B_KO	2	4.34	4.26–93.17	88.91
Log_Males_Mean_SB2_B_KO	4	8.51	2.6–18.81	16.21
Log_Males_Mean_SB2_B_KO	5	42.58	42.28–85.21	42.93
Log_Males_Mean_SB2_B_KO	7	19.28	13.98–61.94	47.97
Log_Males_Mean_SB2_B_KO	11	8.65	8.36–8.67	0.31
Log_Males_Mean_SB2_B_KO	X	22.38	22.37–45.84	23.47
Log_Males_Mean_SB3_A_KO	1	29.17	29.15–31.93	2.78
Log_Males_Mean_SB3_A_KO	2	60.29	60.23–60.29	0.06
Log_Males_Mean_SB3_A_KO	3	37.79	37.79–37.81	0.03
Log_Males_Mean_SB3_A_KO	7	75.12	75.11–75.13	0.02
Log_Males_Mean_SB3_A_KO	9	66.71	66.54–66.73	0.19
Log_Males_Mean_SB3_A_KO	10	36.72	36.72–36.74	0.01
Log_Males_Mean_SB3_A_KO	12	0.16	0.12–18.03	17.91

Log_Males_Mean_SB3_A_KO	14	26.89	26.44–27.24	0.79
Log_Males_Mean_SB3_A_KO	15	49.83	49.77–50.78	1.01
Log_Males_Mean_SB3_A_KO	16	23.2	20.71–23.37	2.66
Log_Males_Mean_SB3_A_KO	18	35.33	35.33–35.34	0.01
Log_Males_Mean_SB3_A_KO	19	45.82	35.48–45.84	10.37
Log_Males_Mean_SB3_A_KO	X	71.04	71.04–71.04	0.0
Log_Males_Mean_SB3_B_KO	2	80.19	45.33–80.26	34.93
Log_Males_Mean_SB3_B_KO	4	18.62	18.6–18.63	0.03
Log_Males_Mean_SB3_B_KO	8	29.2	27.61–29.2	1.59
Log_Males_Mean_SB3_B_KO	10	23.08	22.73–23.93	1.2
Log_Males_Mean_SB3_B_KO	12	6.01	6.0–6.58	0.59
Log_Males_Mean_SB3_B_KO	15	47.78	44.56–48.87	4.31
Log_Males_Mean_SB3_B_KO	16	40.49	40.47–40.55	0.08
Log_Males_Mean_SB3_B_KO	18	53.67	53.66–56.66	3.0
Log_Males_Mean_Small_Intestine_A_KO	1	62.1	62.09–62.17	0.08
Log_Males_Mean_Small_Intestine_A_KO	2	1.25	1.21–1.44	0.23

Log_Males_Mean_Small_Intestine_A_KO	3	45.19	45.15–45.24	0.09
Log_Males_Mean_Small_Intestine_A_KO	7	75.53	66.04–75.57	9.53
Log_Males_Mean_Small_Intestine_A_KO	8	14.91	14.91–14.91	0.0
Log_Males_Mean_Small_Intestine_A_KO	10	75.29	75.29–75.85	0.56
Log_Males_Mean_Small_Intestine_A_KO	12	13.3	13.25–13.37	0.12
Log_Males_Mean_Small_Intestine_A_KO	16	20.51	20.5–20.57	0.07
Log_Males_Mean_Small_Intestine_A_KO	18	48.88	48.86–49.01	0.15
Log_Males_Mean_Small_Intestine_A_KO	X	47.49	12.94–67.17	54.23

Supplementary Table S3: QTL Detected at 0.5 LOD Threshold for Polyp Development across Different Intestinal Segments (female population)

Detailed presentation of QTL across chromosomes 1, 2, 3, 7, and 15 for polyp development in the small intestine and colon, with corresponding confidence intervals.

lodcolumn	chr	Peak (Mb)	95% CI (Mb)	Size (Mb)
Females_Mean_SB1_A_KO	8	16.49	15.26–17.31	2.05
Females_Mean_SB1_C_KO	1	46.35	46.35–46.35	0.0
Females_Mean_SB1_C_KO	2	52.08	50.67–53.11	2.44
Females_Mean_SB1_C_KO	4	77.31	60.45–77.4	16.95
Females_Mean_SB1_C_KO	8	60.61	51.68–60.72	9.04
Females_Mean_SB2_B_KO	1	65.09	1.22–66.48	65.25
Females_Mean_SB2_B_KO	5	19.02	18.92–19.19	0.28
Females_Mean_SB2_B_KO	6	35.11	34.85–38.61	3.76
Females_Mean_SB2_B_KO	9	12.6	12.56–12.6	0.04
Females_Mean_SB2_B_KO	11	60.97	59.8–61.21	1.41
Females_Mean_SB2_B_KO	15	52.17	51.6–54.47	2.87
Females_Mean_SB2_B_KO	X	9.09	9.02–60.4	51.37
Females_Mean_SB2_C_KO	1	3.05	2.35–3.1	0.75

Females_Mean_SB2_C_KO	3	79.93	79.83–79.96	0.13
Females_Mean_SB2_C_KO	4	13.25	13.2–19.91	6.71
Females_Mean_SB2_C_KO	6	22.76	22.46–22.89	0.43
Females_Mean_SB2_C_KO	7	52.14	52.08–86.27	34.19
Females_Mean_SB2_C_KO	8	48.58	46.82–67.52	20.71
Females_Mean_SB2_C_KO	11	34.18	34.05–34.18	0.12
Females_Mean_SB2_C_KO	19	18.25	1.55–18.25	16.7
Females_Mean_SB3_C_KO	1	46.35	46.35–49.51	3.16
Females_Mean_SB3_C_KO	2	52.55	50.5–52.55	2.05
Females_Mean_SB3_C_KO	6	2.83	2.83–2.83	0.0
Females_Mean_SB3_C_KO	7	11.78	11.78–11.78	0.0
Females_Mean_SB3_C_KO	8	59.75	51.67–61.14	9.47
Females_Mean_SB3_C_KO	10	12.58	12.57–12.58	0.01
Females_Mean_SB3_C_KO	12	51.67	41.89–51.74	9.85
Females_Mean_SB3_C_KO	15	15.31	15.31–15.31	0.0
Females_Mean_SB3_KO	12	51.67	43.82–58.39	14.57

Females_Mean_Small_Intestine_C_KO	2	52.08	52.08–52.08	0.0
Females_Mean_Small_Intestine_C_KO	3	21.08	20.26–25.87	5.6
Females_Mean_Small_Intestine_C_KO	4	77.31	6.29–77.4	71.11
Females_Mean_Small_Intestine_C_KO	6	22.02	2.83–22.25	19.42
Females_Mean_Small_Intestine_C_KO	12	42.7	37.77–45.28	7.51
Females_Mean_Small_Intestine_C_KO	19	20.27	20.24–20.47	0.23
Females_Mean_Total_Small_Intestine_KO	1	46.35	8.75–48.32	39.57
Females_Mean_Total_Small_Intestine_KO	2	52.08	52.08–54.33	2.24
Females_Mean_Total_Small_Intestine_KO	4	6.3	6.29–6.31	0.02
Females_Mean_Total_Small_Intestine_KO	6	2.83	2.83–22.06	19.23
Females_Mean_Total_Small_Intestine_KO	8	58.75	58.71–59.82	1.11
Females_Mean_Total_Small_Intestine_KO	12	45.28	37.77–47.67	9.9
Females_Mean_Total_Small_Intestine_KO	19	38.89	31.73–39.12	7.39
Females_Mean_Total_Small_Intestine_KO	X	3.29	3.29–3.29	0.0
Females_Mean_Colon_A_KO	1	64.14	64.14–64.15	0.01
Females_Mean_Colon_A_KO	2	57.9	57.89–57.9	0.0

Females_Mean_Colon_A_KO	5	2.26	2.24–2.27	0.03
Females_Mean_Colon_A_KO	7	82.37	82.34–82.47	0.13
Females_Mean_Colon_A_KO	11	57.07	57.07–57.08	0.01
Females_Mean_Colon_A_KO	15	6.48	6.47–6.58	0.11
Females_Mean_Colon_A_KO	18	49.45	49.34–49.49	0.15
Females_Mean_Colon_C_KO	8	58.63	58.1–60.69	2.59
Females_Mean_Colon_C_KO	12	39.11	39.02–39.13	0.11
Females_Mean_Total_Colon_KO	3	21.33	21.12–34.35	13.22
Females_Mean_Total_Colon_KO	8	60.61	58.1–60.69	2.59
Females_Mean_Total_Colon_KO	12	39.11	39.03–39.13	0.1
Females_Mean_Total_Intestinal_KO	1	46.35	6.88–46.35	39.47
Females_Mean_Total_Intestinal_KO	2	55.41	55.3–55.48	0.18
Females_Mean_Total_Intestinal_KO	3	34.01	33.88–34.05	0.17
Females_Mean_Total_Intestinal_KO	6	3.86	3.86–4.08	0.22
Females_Mean_Total_Intestinal_KO	8	60.42	51.18–60.69	9.51
Females_Mean_Total_Intestinal_KO	10	51.83	51.83–52.01	0.18

Females_Mean_Total_Intestinal_KO	12	45.28	45.28–45.28	0.0
Females_Mean_Total_Intestinal_KO	19	38.45	37.79–38.98	1.19
Log_Females_Mean_SB2_B_KO	1	65.09	65.09–66.48	1.39
Log_Females_Mean_SB2_B_KO	5	19.02	18.94–19.19	0.26
Log_Females_Mean_SB2_B_KO	11	59.8	59.8–61.02	1.22
Log_Females_Mean_SB2_B_KO	14	19.49	19.45–19.5	0.06
Log_Females_Mean_SB2_B_KO	15	52.17	52.03–54.47	2.45
Log_Females_Mean_SB2_B_KO	X	61.07	46.62–61.55	14.93
Log_Females_Mean_SB2_C_KO	3	79.93	79.87–79.96	0.09
Log_Females_Mean_SB2_C_KO	7	52.14	52.11–86.27	34.16
Log_Females_Mean_SB2_C_KO	11	66.03	66.0–66.04	0.04
Log_Females_Mean_SB2_C_KO	15	27.77	1.53–27.81	26.28
Log_Females_Mean_SB2_C_KO	19	2.56	2.51–2.58	0.07
Log_Females_Mean_SB3_A_KO	3	78.59	78.54–78.64	0.1
Log_Females_Mean_SB3_B_KO	1	90.84	85.27–93.53	8.26
Log_Females_Mean_SB3_B_KO	7	74.45	74.45–74.46	0.01

Log_Females_Mean_SB3_B_KO	12	8.52	8.45–8.55	0.1
Log_Females_Mean_SB3_B_KO	18	32.77	32.77–32.87	0.1
Log_Females_Mean_SB3_C_KO	1	49.5	46.35–53.42	7.07
Log_Females_Mean_SB3_C_KO	2	53.55	53.52–53.58	0.06
Log_Females_Mean_SB3_C_KO	6	2.83	2.83–2.83	0.0
Log_Females_Mean_SB3_C_KO	10	12.58	12.57–12.58	0.01
Log_Females_Mean_SB3_C_KO	12	46.21	46.21–57.92	11.71
Log_Females_Mean_SB3_C_KO	15	15.31	15.31–15.31	0.0
Log_Females_Mean_SB3_KO	12	57.92	44.3–58.42	14.13
Log_Females_Mean_SB3_KO	17	17.37	17.37–17.37	0.0
Log_Females_Mean_Small_Intestine_C_KO	2	53.72	52.08–53.95	1.87
Log_Females_Mean_Small_Intestine_C_KO	3	20.44	2.98–34.01	31.02
Log_Females_Mean_Small_Intestine_C_KO	4	6.3	6.29–77.31	71.02
Log_Females_Mean_Small_Intestine_C_KO	6	22.35	7.01–22.35	15.34
Log_Females_Mean_Small_Intestine_C_KO	10	7.92	3.37–52.23	48.86
Log_Females_Mean_Small_Intestine_C_KO	12	45.28	45.28–45.28	0.0

Log_Females_Mean_Small_Intestine_C_KO	13	49.1	49.1–49.1	0.0
Log_Females_Mean_Small_Intestine_C_KO	14	45.07	45.07–45.07	0.0
Log_Females_Mean_Small_Intestine_C_KO	15	6.02	5.95–6.02	0.07
Log_Females_Mean_Small_Intestine_C_KO	16	39.5	38.96–40.49	1.53
Log_Females_Mean_Small_Intestine_C_KO	17	17.37	17.37–46.24	28.88
Log_Females_Mean_Total_Small_Intestine_KO	2	54.06	52.08–66.13	14.05
Log_Females_Mean_Total_Small_Intestine_KO	4	6.3	6.29–6.31	0.02
Log_Females_Mean_Total_Small_Intestine_KO	6	2.83	2.83–4.08	1.25
Log_Females_Mean_Total_Small_Intestine_KO	8	58.75	58.71–58.77	0.06
Log_Females_Mean_Total_Small_Intestine_KO	11	43.96	43.96–43.96	0.01
Log_Females_Mean_Total_Small_Intestine_KO	13	49.1	49.1–49.1	0.0
Log_Females_Mean_Total_Small_Intestine_KO	19	38.97	36.99–39.07	2.08
Log_Females_Mean_Total_Small_Intestine_KO	X	3.29	3.29–3.29	0.0
Log_Females_Mean_Total_Intestinal_KO	1	8.51	8.5–46.35	37.85
Log_Females_Mean_Total_Intestinal_KO	3	34.01	33.88–34.05	0.17
Log_Females_Mean_Total_Intestinal_KO	8	60.18	27.29–60.25	32.96

Log_Females_Mean_Total_Intestinal_KO	16	39.42	39.36–39.6	0.24
Log_Females_Mean_Total_Intestinal_KO	19	38.45	37.79–38.55	0.76

Supplementary Table S4.

Significant transcription factor enrichments from ChEA 2022.

Candidate genes within QTL intervals were tested against the ChEA 2022 database in Enrichr. Transcription factors with **FDR-adjusted p-values < 0.05** were considered statistically significant, and those with **0.05 ≤ FDR < 0.1** were considered suggestive. The table lists enriched transcription factors, adjusted p-values, and associated enrichment statistics.

	term	adjusted p-value
0	FLI1 27457419 Chip-Seq LIVER Mouse	3.96E-257
1	PCGF2 27294783 Chip-Seq ESCs Mouse	8.42E-221
2	SUZ12 27294783 Chip-Seq NPCs Mouse	2.76E-156
3	NFE2 27457419 Chip-Seq LIVER Mouse	4.55E-149
4	PCGF2 27294783 Chip-Seq NPCs Mouse	1.42E-104
5	EZH2 27294783 Chip-Seq NPCs Mouse	1.55E-104
6	RUNX1 27457419 Chip-Seq LIVER Mouse	4.81E-73
7	EBF1 22473956 ChIP-Seq LYMPHODE Mouse	1.48E-72
8	EBF1 22473956 ChIP-Seq BONE MARROW Mouse	4.88E-71
9	PU1 27457419 Chip-Seq LIVER Mouse	7.12E-70
10	P53 22387025 ChIP-Seq ESCs Mouse	7.72E-70
11	SMAD1 18555785 Chip-Seq ESCs Mouse	8.68E-64
12	CDX2 21402776 ChIP-Seq INTESTINAL-VILLUS Mouse	2.68E-63
13	CRX 20693478 ChIP-Seq RETINA Mouse	3.34E-63
14	SOX6 21985497 ChIP-Seq MYOTUBES Mouse	6.12E-63
15	OCT4 21477851 ChIP-Seq ESCs Mouse	1.76E-61
16	ZFX 18555785 Chip-Seq ESCs Mouse	3.66E-58
17	OCT4 18555785 Chip-Seq ESCs Mouse	1.58E-55
18	P300 18555785 Chip-Seq ESCs Mouse	4.37E-55
19	CTCF 18555785 Chip-Seq ESCs Mouse	4.94E-55
20	KLF4 18555785 Chip-Seq ESCs Mouse	6.50E-55
21	STAT3 18555785 Chip-Seq ESCs Mouse	2.44E-54
22	NMYC 18555785 Chip-Seq ESCs Mouse	2.21E-53
23	ESRRB 18555785 Chip-Seq ESCs Mouse	1.02E-52
24	SOX2 18555785 Chip-Seq ESCs Mouse	5.76E-52
25	SUZ12 18555785 Chip-Seq ESCs Mouse	5.69E-51

26	CMYC 18555785 Chip-Seq ESCs Mouse	1.75E-50
27	E2F1 18555785 Chip-Seq ESCs Mouse	3.10E-44
28	NANOG 18555785 Chip-Seq ESCs Mouse	1.65E-33
29	TCFCP2L1 18555785 Chip-Seq ESCs Mouse	3.09E-32
30	RBPJ 22232070 ChIP-Seq NCS Mouse	1.60E-21
31	PU 27001747 Chip-Seq BMDM Mouse	1.85E-13
32	SOX9 26525672 Chip-Seq Limbbuds Mouse	2.65E-08
33	DPY 21335234 ChIP-Seq ESCs Mouse	0.000514
34	ASXL1 24218140 ChIP-Seq BMDM Mouse	0.053342

Supplementary Table S5.

Significant transcription factor enrichments from Rummagene.

Candidate genes within QTL intervals were tested against the Rummagene transcription factor co-regulation database in Enrichr. Transcription factors meeting the same thresholds (FDR < 0.05 significant; 0.05 ≤ FDR < 0.1 suggestive) are reported. The table provides enriched transcription factors, adjusted p-values, and associated enrichment statistics.

	term	adjusted p-value
0	PMC7592539-12885 2020 7456 MOESM4 ESM.xlsx-Sup TableS3 LMS Del Genes Conf-2Q37 1 TP63	2.99E-105
1	PMC2742312-1479-7364-3-3-221-S3.PDF-64-ALS2CR8 CARF	1.89E-27
2	PMC8046804-41467 2021 22478 MOESM11 ESM.xlsx-Del AA-TCGA-Unnamed 36 TEAD1	3.26E-11
3	PMC6263041-12883 2018 1199 MOESM3 ESM.xlsx-Sheet1-SIX3 SIX3	0.001567
4	PMC8046804-41467 2021 22478 MOESM11 ESM.xlsx-Del AA-TCGA-Unnamed 35 TEAD1	0.011232

Supplementary Figure S1. QTL Mapping Results – Full Cohort

LOD profile showing the QTL identified on **chromosome 14** for colon polyp burden in the full cohort (`Log_Mean_Colon_B_KO`). A significant peak was observed at ~33 Mb, marking a locus associated with polyp susceptibility.

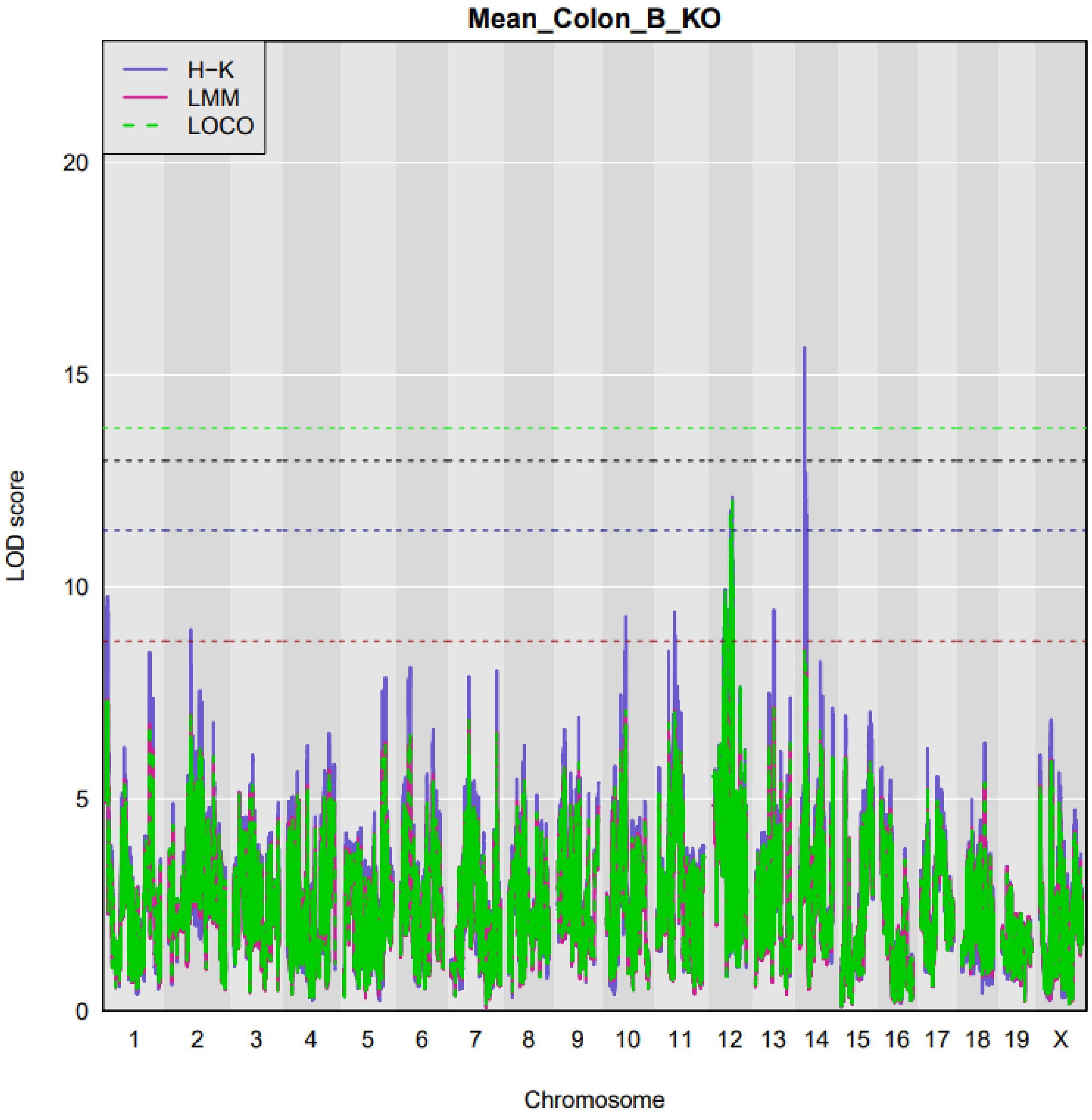

Supplementary Figure S2. Founder Allele Effects – Full Cohort

Founder strain allele effects for the QTL on **chromosome 14**, corresponding to the full cohort analysis (`Log_Mean_Colon_B_KO`). CAST and WSB alleles were among those showing large effect sizes at the peak locus.

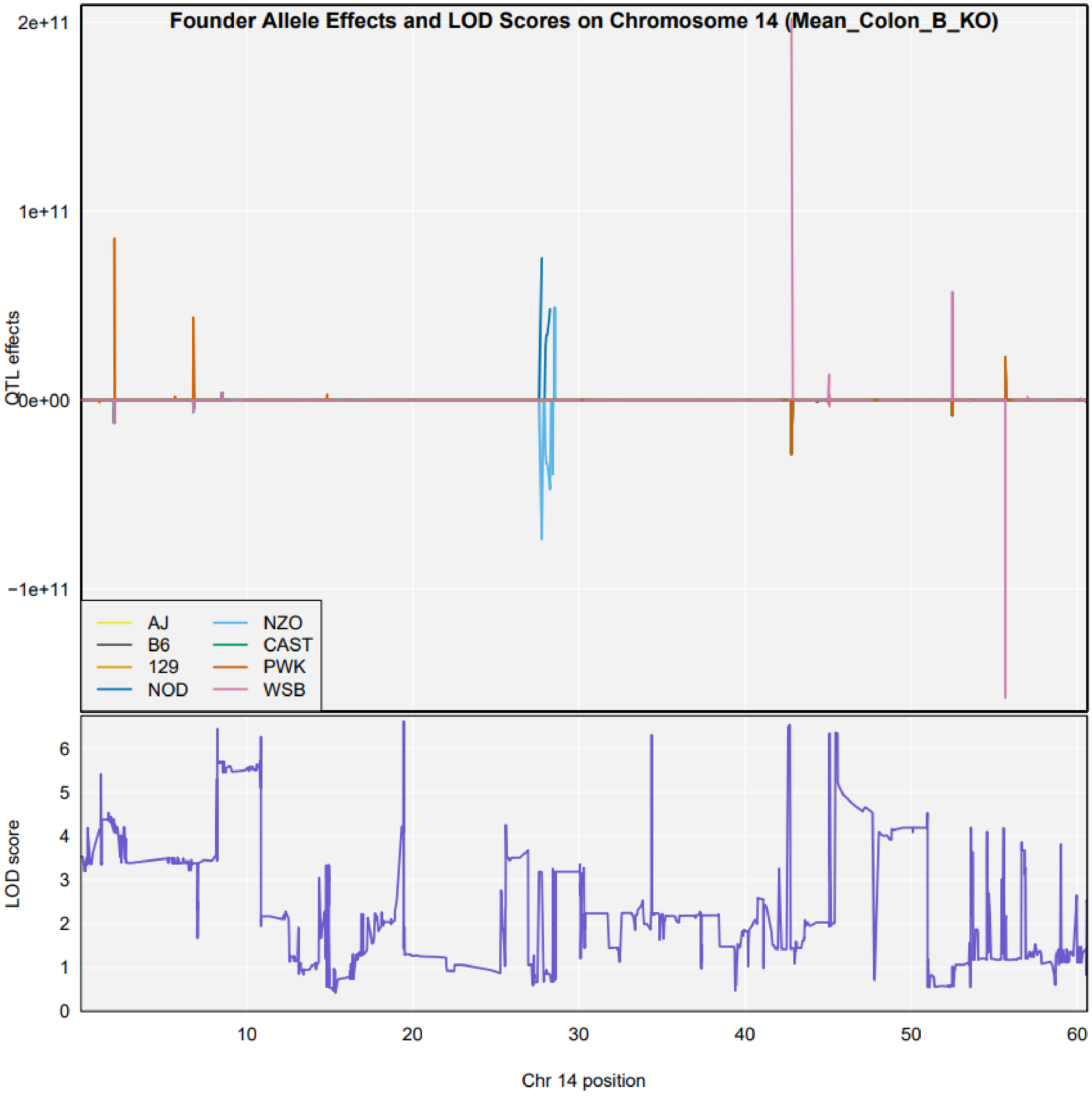

Supplementary Figure S3. QTL Mapping Results – Male Subset

(A) QTL on **chromosome 1** for SB1 polyp count (`Males_Mean_SB1_A_KO`).
(B) QTL on **chromosome 3** for total small intestinal burden (`Males_Mean_Small_Intestine_A_KO`).
(C) Replicated QTL on **chromosome1 and 15** in log-transformed SB1 data (`Log_Males_Mean_SB1_A_KO`), supporting signal robustness.

(A)

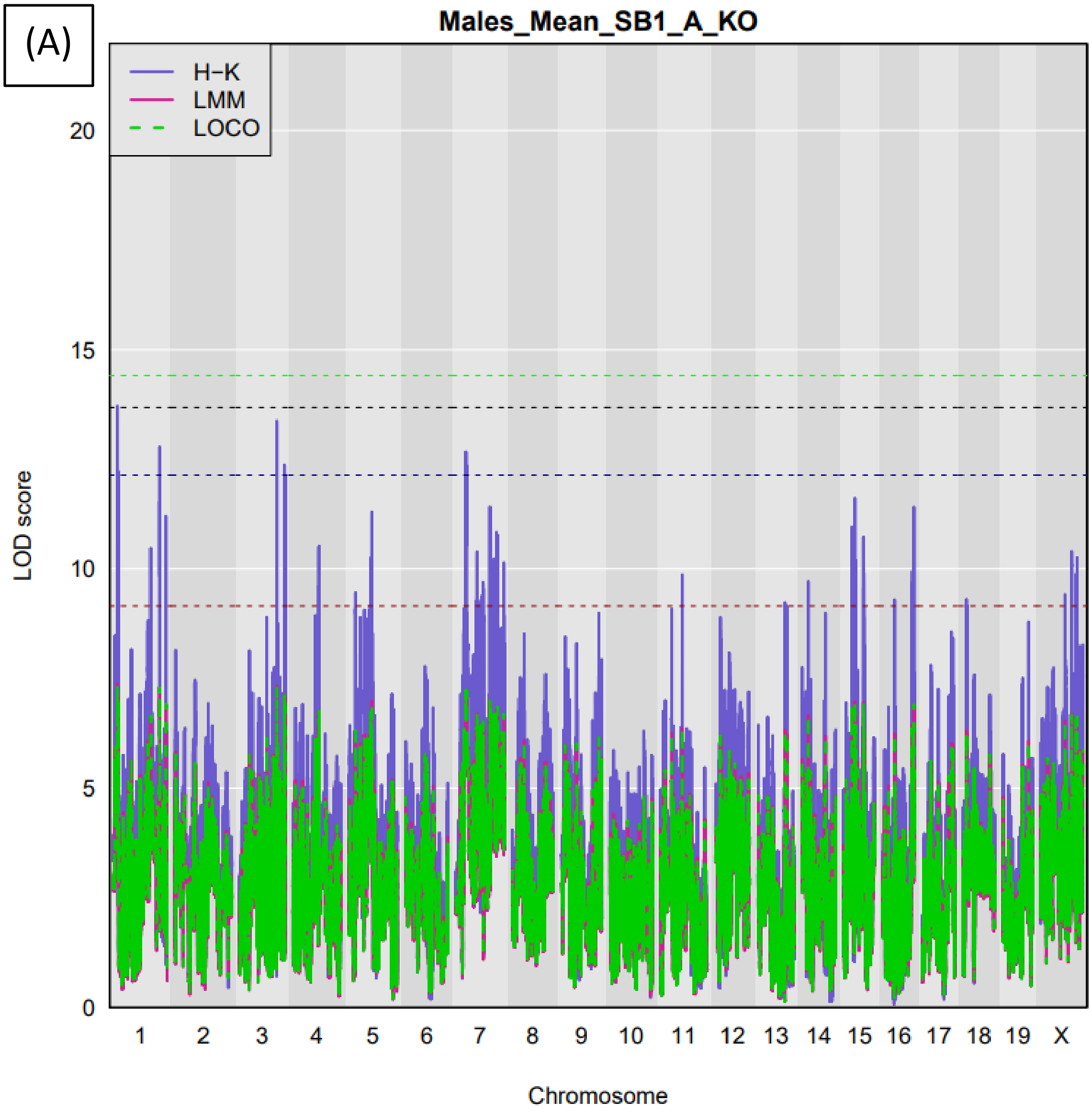

(B)

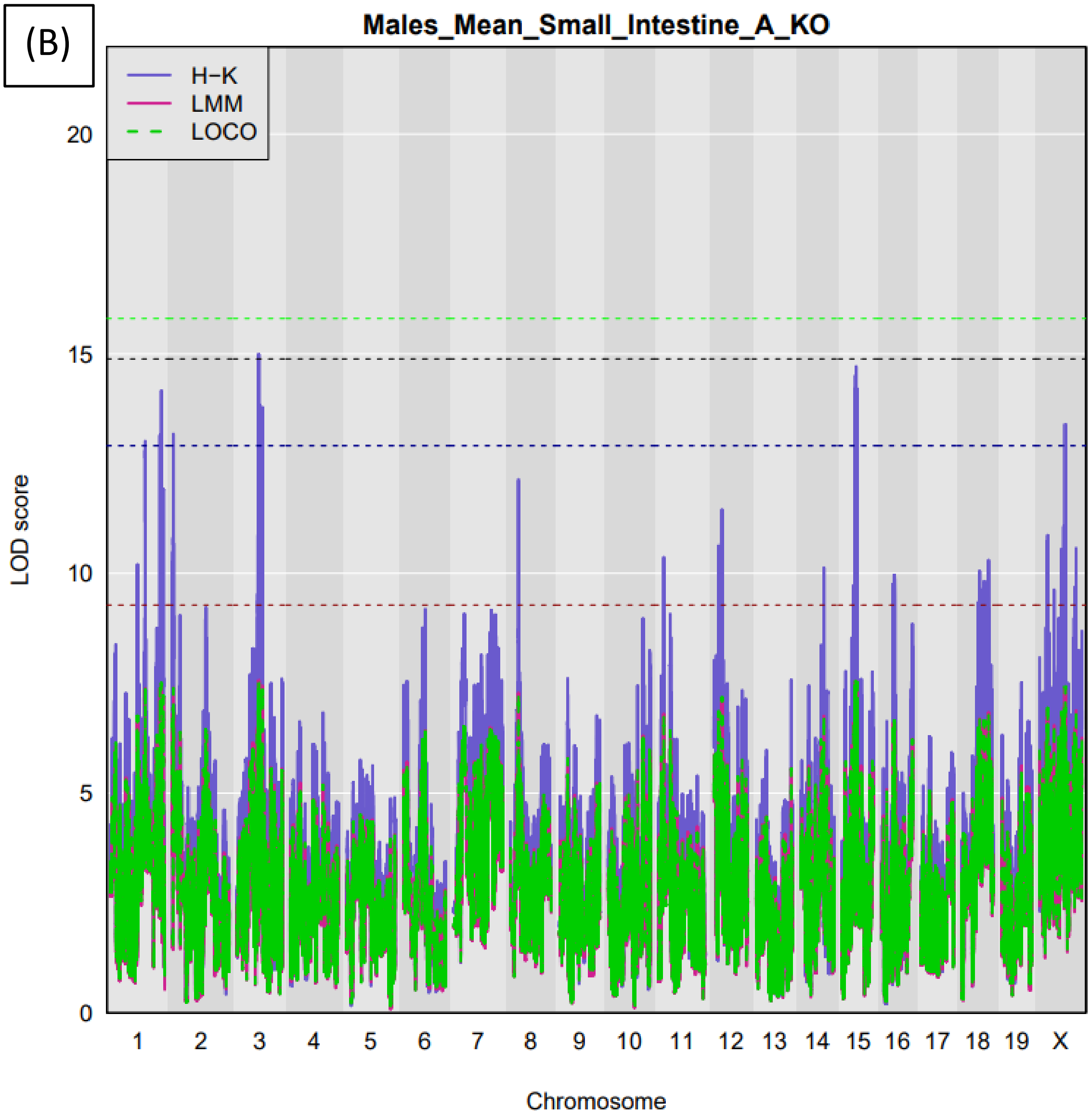
Males_Mean_Small_Intestine_A_KO
H−K
LMM
LOCO
LOD score
0
5
10
15
20
1 2 3 4 5 6 7 8 9 10 11 12 13 14 15 16 17 18 19 X
Chromosome

(C)

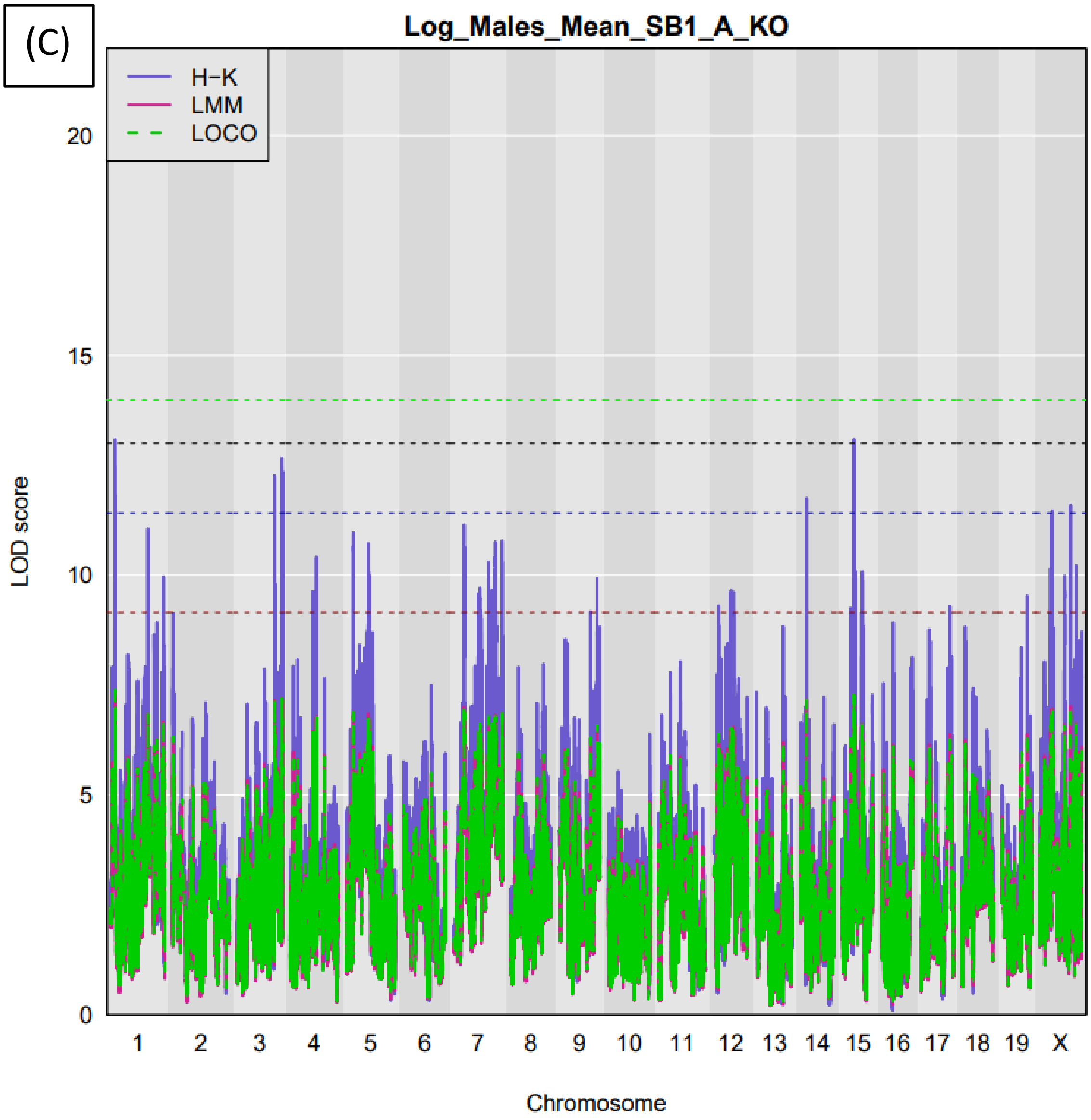
Log_Males_Mean_SB1_A_KO
H−K
LMM
LOCO
LOD score
0
5
10
15
20
1 2 3 4 5 6 7 8 9 10 11 12 13 14 15 16 17 18 19 X
Chromosome

Supplementary Figure S4. Founder Allele Effects – Male Subset

Founder strain effects for male-specific QTLs:
(A) Chromosome 1 – SB1 trait (`Males_Mean_SB1_A_KO`).
(B) Chromosome 3 – total small intestine (`Males_Mean_Small_Intestine_A_KO`).
(C) Chromosome 15 – log-transformed SB1 (`Log_Males_Mean_Small_Intestine_A_KO`).
CAST, PWK, and NOD strains showed the strongest effects in region-specific patterns.

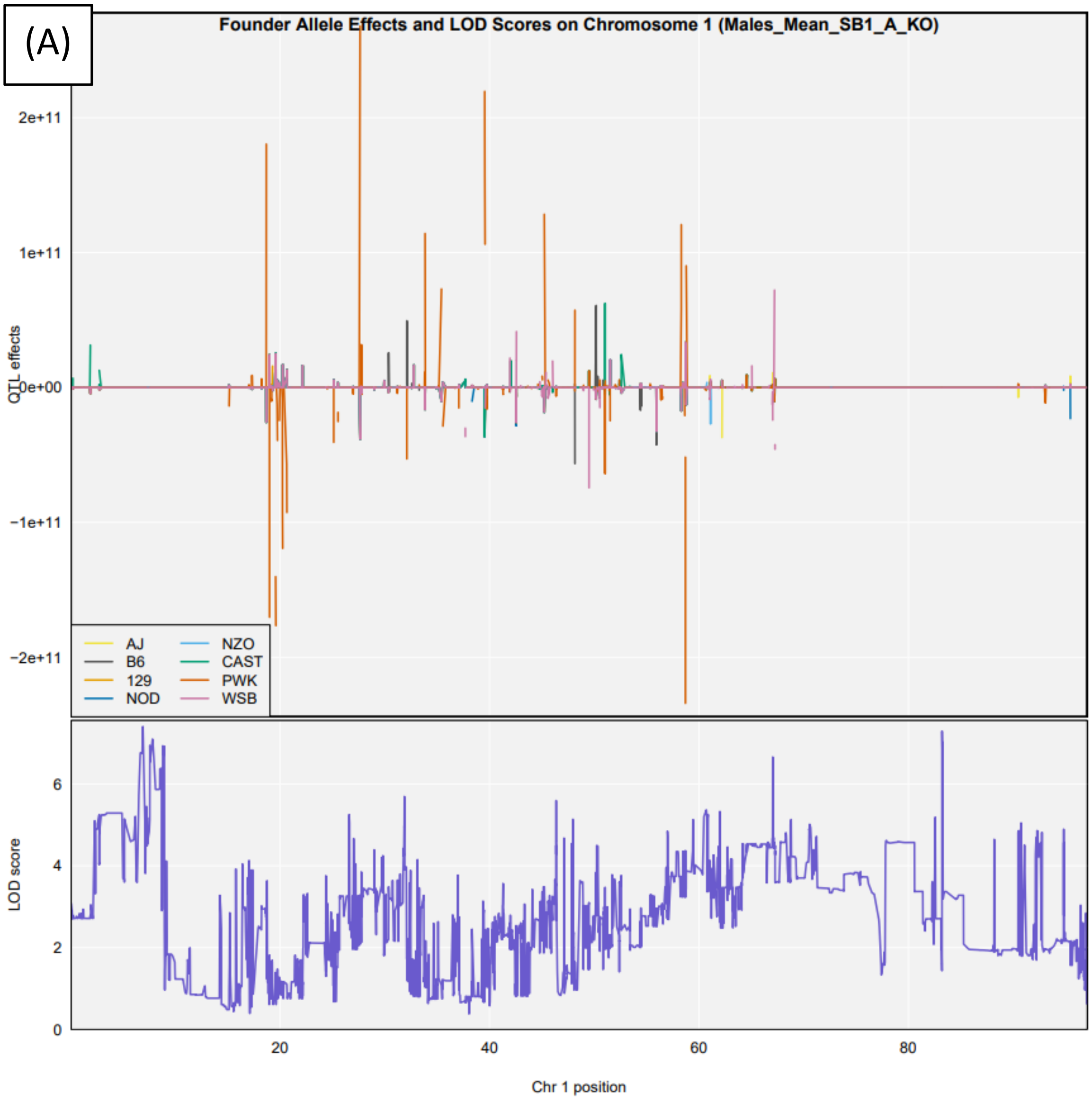

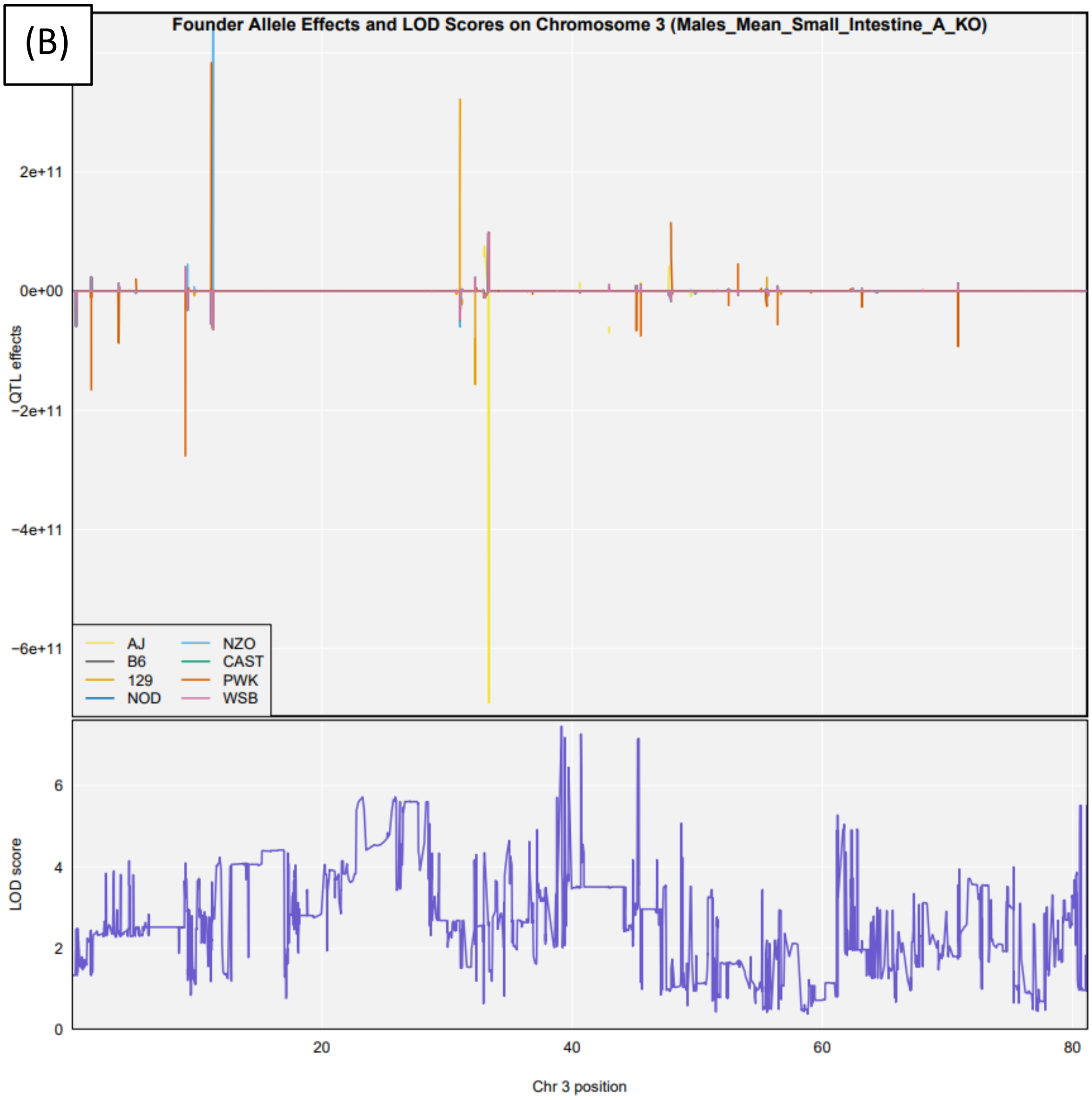

(B)
Founder Allele Effects and LOD Scores on Chromosome 3 (Males_Mean_Small_Intestine_A_KO)
QTL effects
2e+11
0e+00
−2e+11
−4e+11
−6e+11
AJ
B6
129
NOD
NZO
CAST
PWK
WSB
LOD score
6
4
2
0
20
40
60
80
Chr 3 position

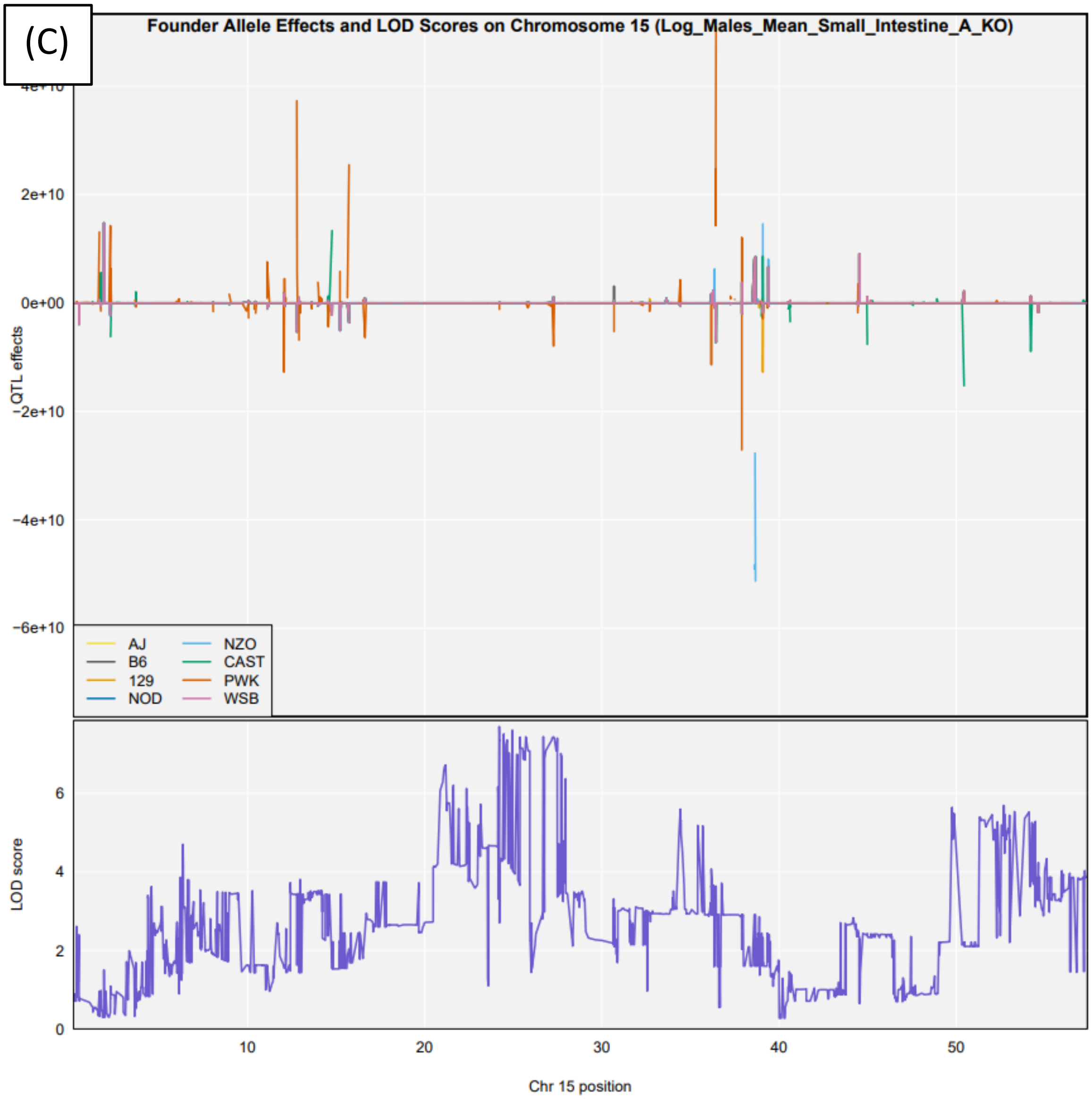
(C)
Founder Allele Effects and LOD Scores on Chromosome 15 (Log_Males_Mean_Small_Intestine_A_KO)
QTL effects
2e+10
0e+00
−2e+10
−4e+10
−6e+10
AJ
B6
129
NOD
NZO
CAST
PWK
WSB
LOD score
6
4
2
0
10
20
30
40
50
Chr 15 position

Supplementary Figure S5. QTL Mapping Results – Female Subset

(A) Chromosome 17 – SB2 (`Females_Mean_SB2_C_KO`).
(B) Chromosome 1 – total small intestine (`Females_Mean_Small_Intestine_C_KO`).
(C) Chromosome 17 – log SB2 (`Log_Females_Mean_SB2_C_KO`).
(D) Chromosome 17 – SB3 (`Log_Females_Mean_SB3_C_KO`).
(E) Chromosome 1 – total small intestine (`Log_Females_Mean_Total_Small_Intestine_KO`).

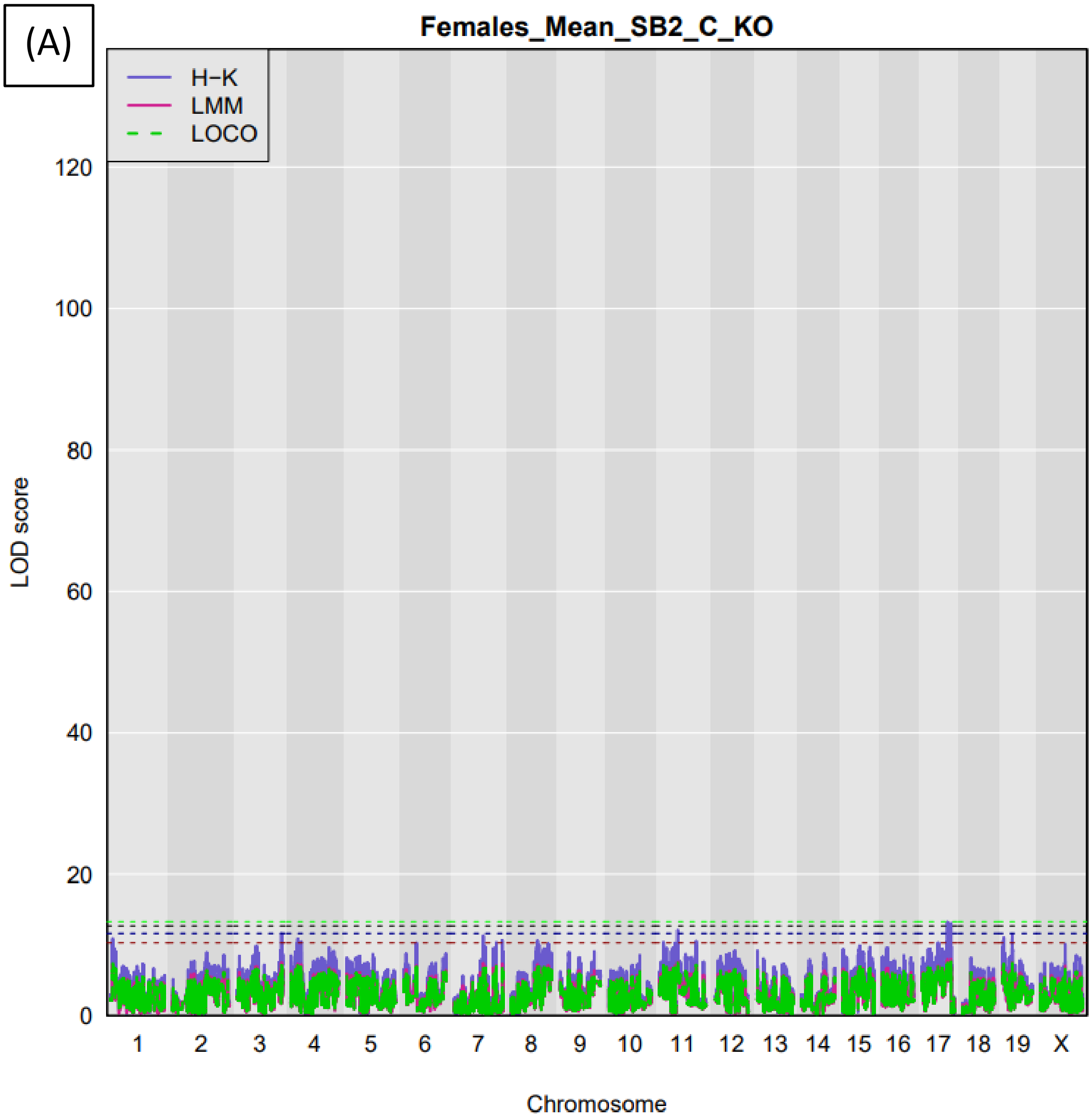

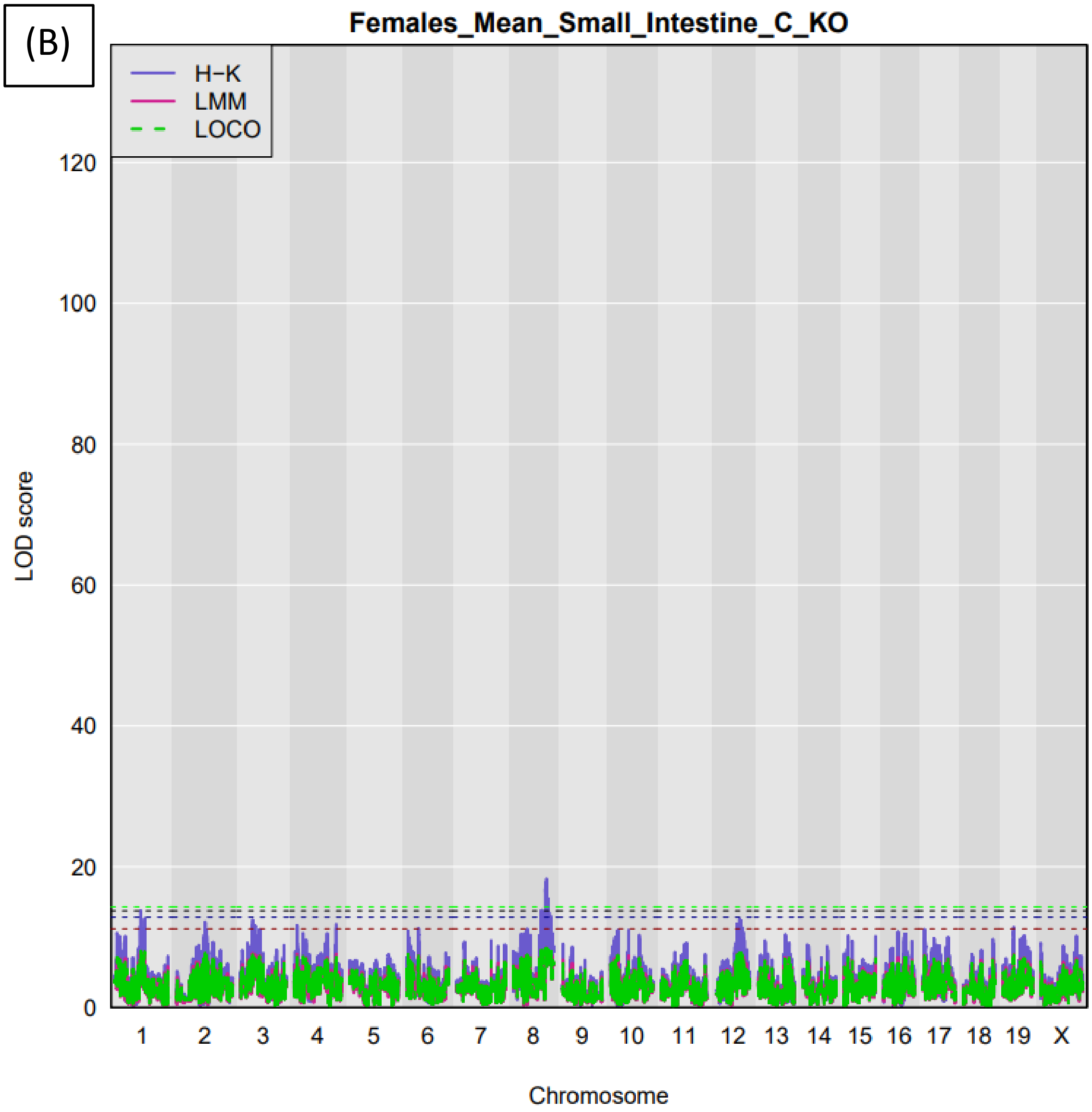
(B)
Females_Mean_Small_Intestine_C_KO
H−K
LMM
LOCO
LOD score
120
100
80
60
40
20
0
1
2
3
4
5
6
7
8
9
10
11
12
13
14
15
16
17
18
19
X
Chromosome

(C)

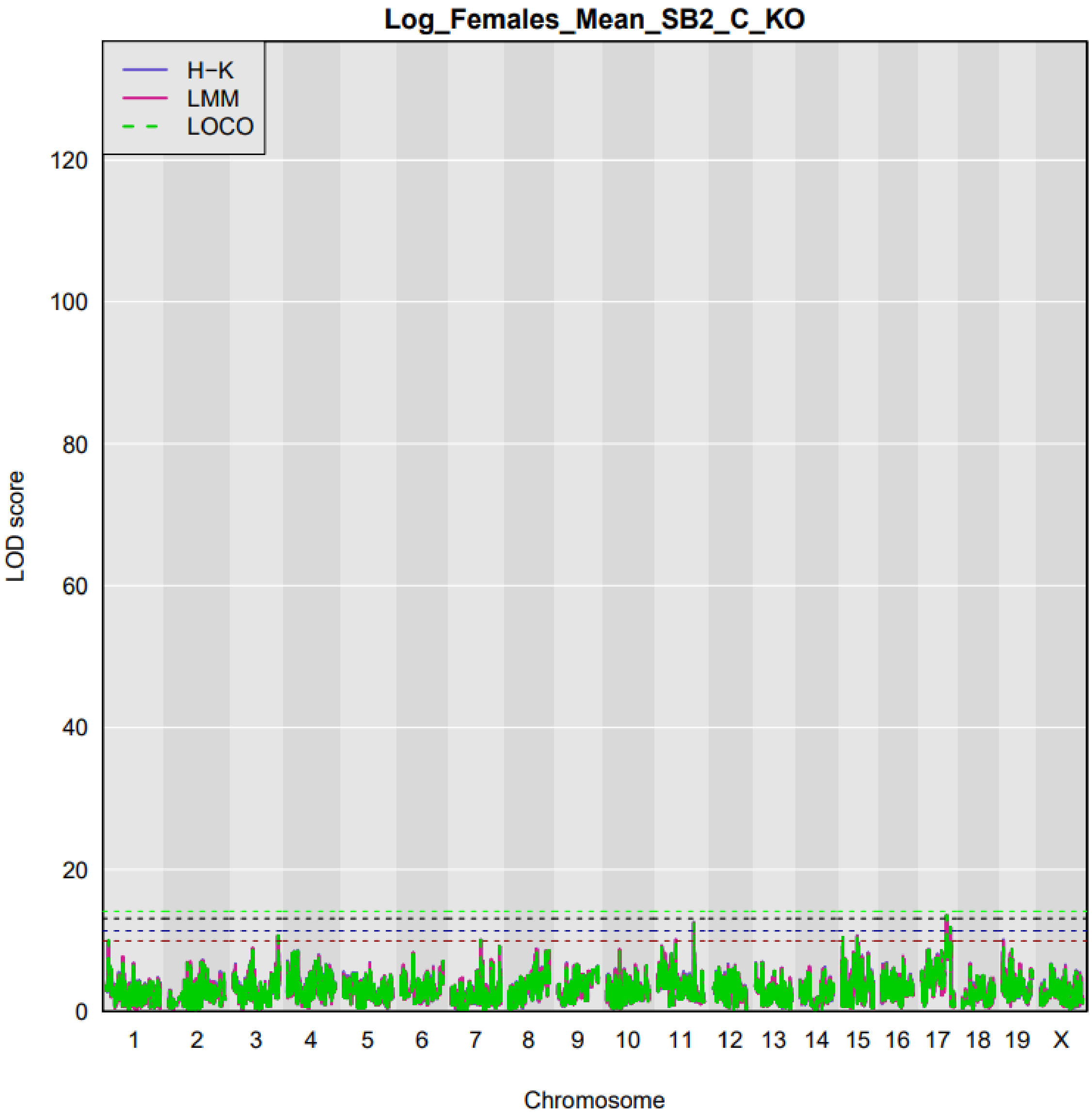
Log_Females_Mean_SB2_C_KO
H−K
LMM
LOCO
LOD score
0
20
40
60
80
100
120
1
2
3
4
5
6
7
8
9
10
11
12
13
14
15
16
17
18
19
X
Chromosome

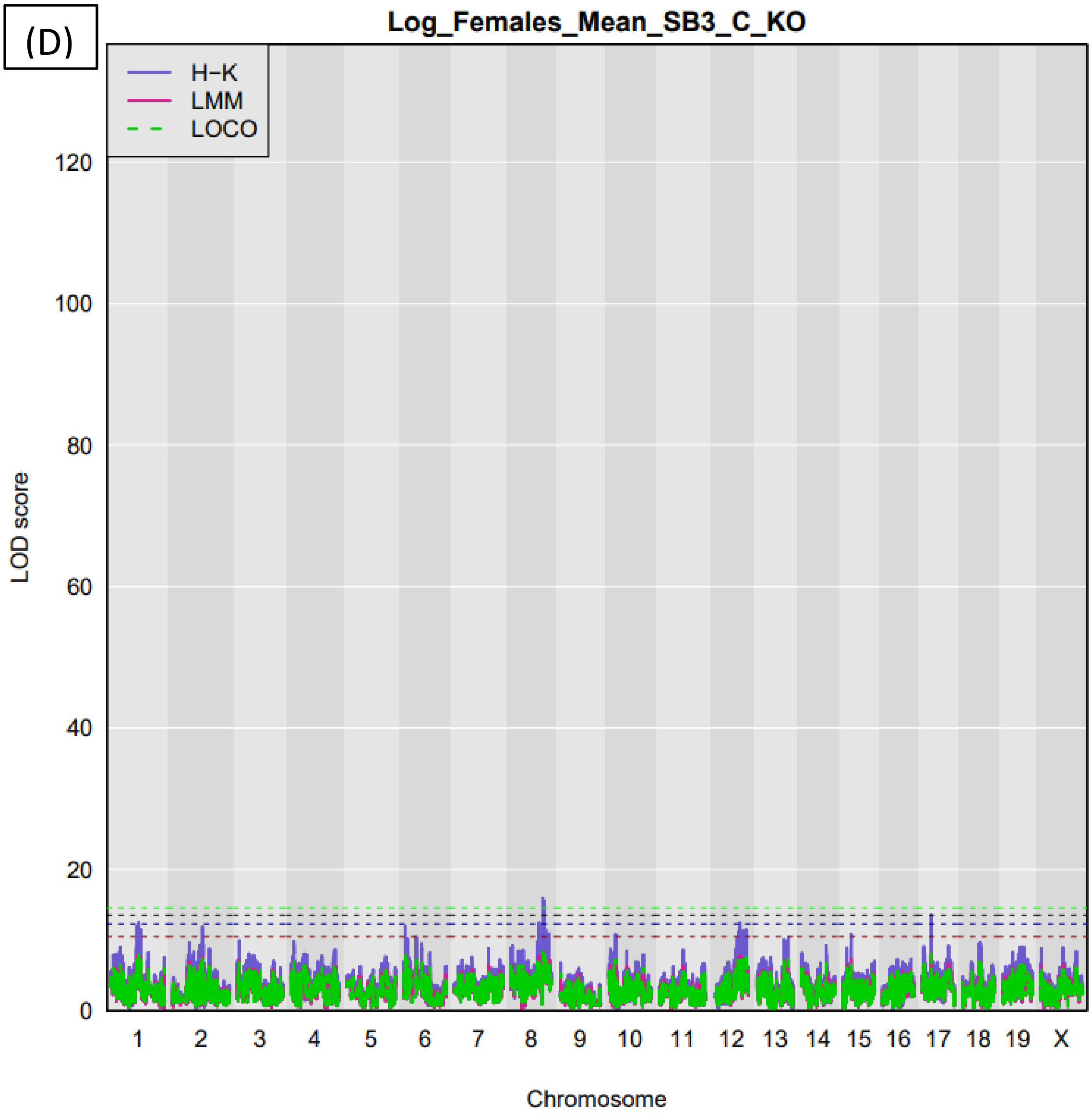
(D)
Log_Females_Mean_SB3_C_KO
H−K
LMM
LOCO
LOD score
0
20
40
60
80
100
120
1
2
3
4
5
6
7
8
9
10
11
12
13
14
15
16
17
18
19
X
Chromosome

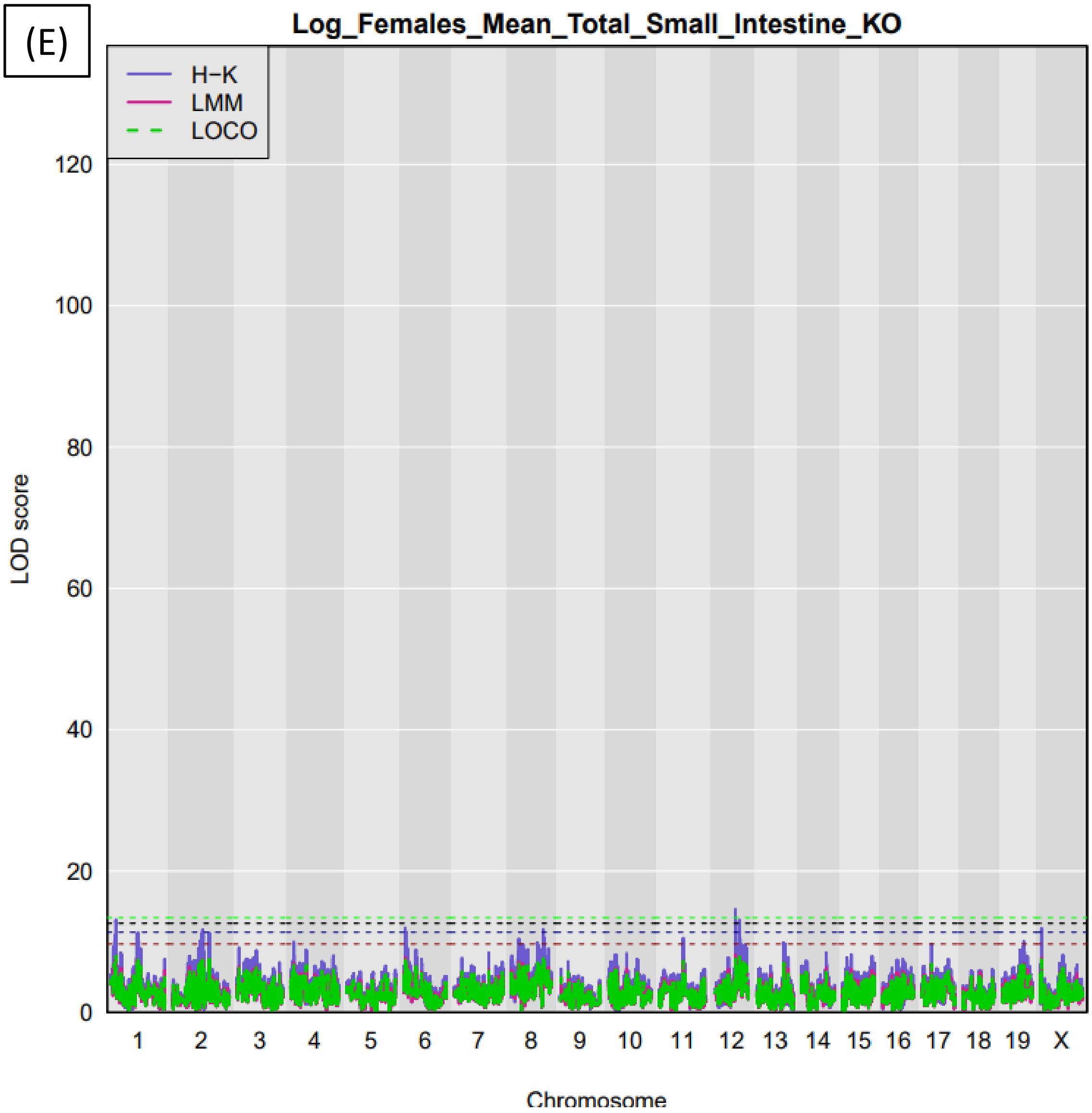

(E)
Log_Females_Mean_Total_Small_Intestine_KO
H−K
LMM
LOCO
LOD score
120
100
80
60
40
20
0
1
2
3
4
5
6
7
8
9
10
11
12
13
14
15
16
17
18
19
X
Chromosome

Supplementary Figure S6. Founder Allele Effects – Female Subset

Founder strain-specific effects for each QTL mapped in the female cohort:
(A) Chromosome 17 – SB2 and SB3.
(B) Chromosome 1 – total SI.
(C) Chromosome 17 – log SB2.
(D) Chromosome 1 – total SI.
PWK, CAST, and B6 alleles displayed prominent contributions to phenotype variance across loci.

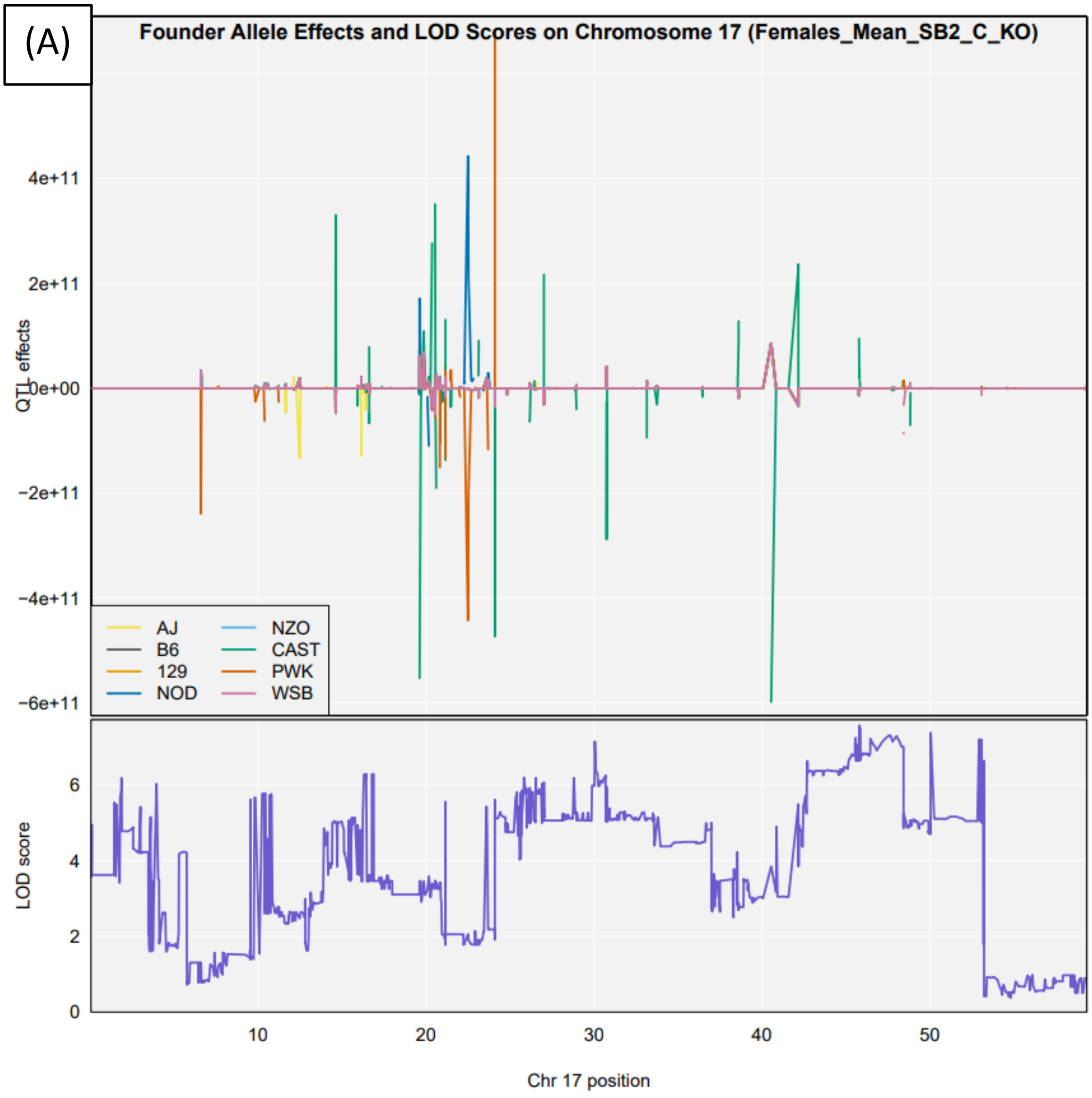

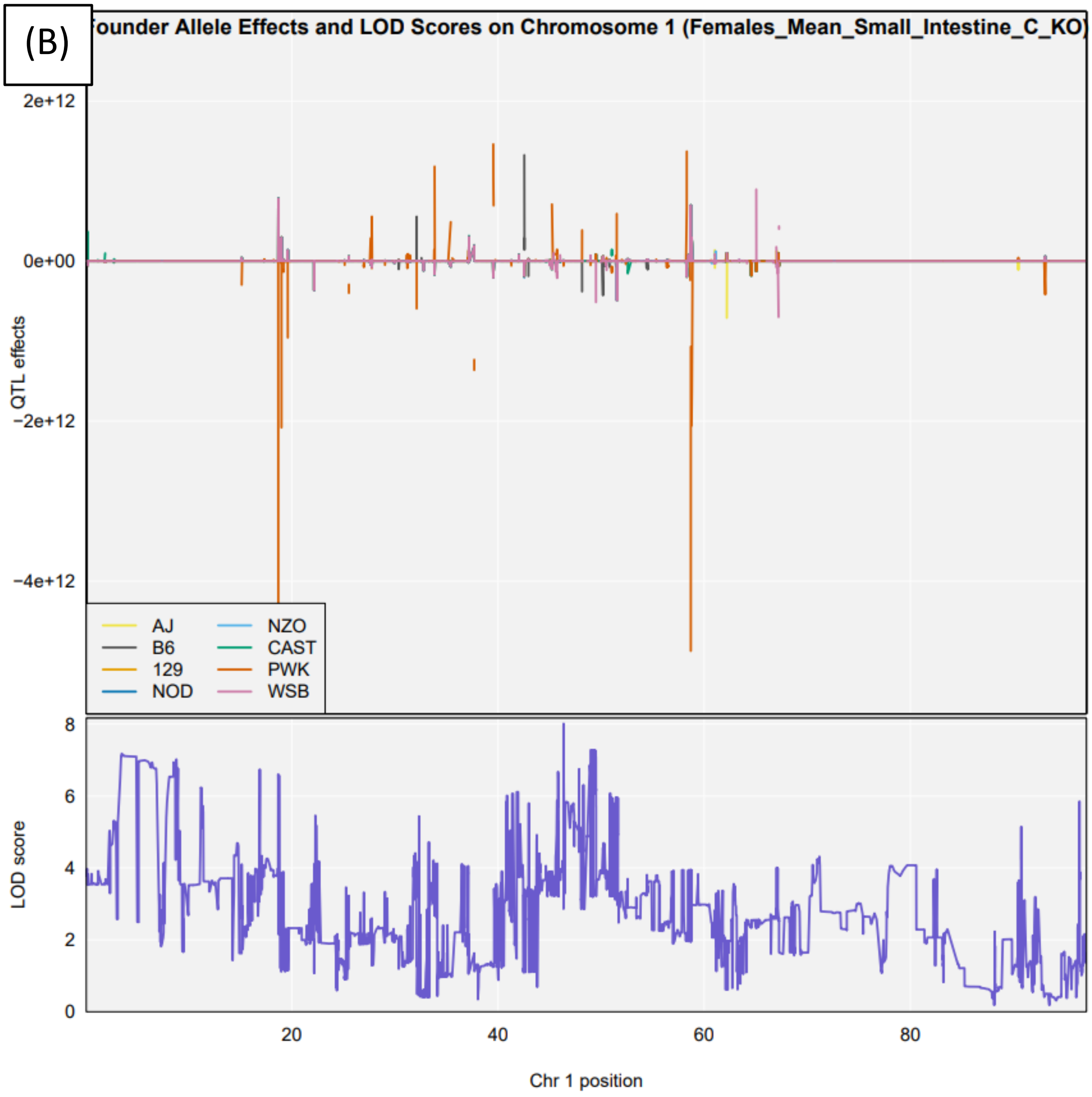
(B)
Founder Allele Effects and LOD Scores on Chromosome 1 (Females_Mean_Small_Intestine_C_KO)
2e+12
0e+00
−2e+12
−4e+12
QTL effects
AJ
B6
129
NOD
NZO
CAST
PWK
WSB
8
6
4
2
0
LOD score
20
40
60
80
Chr 1 position

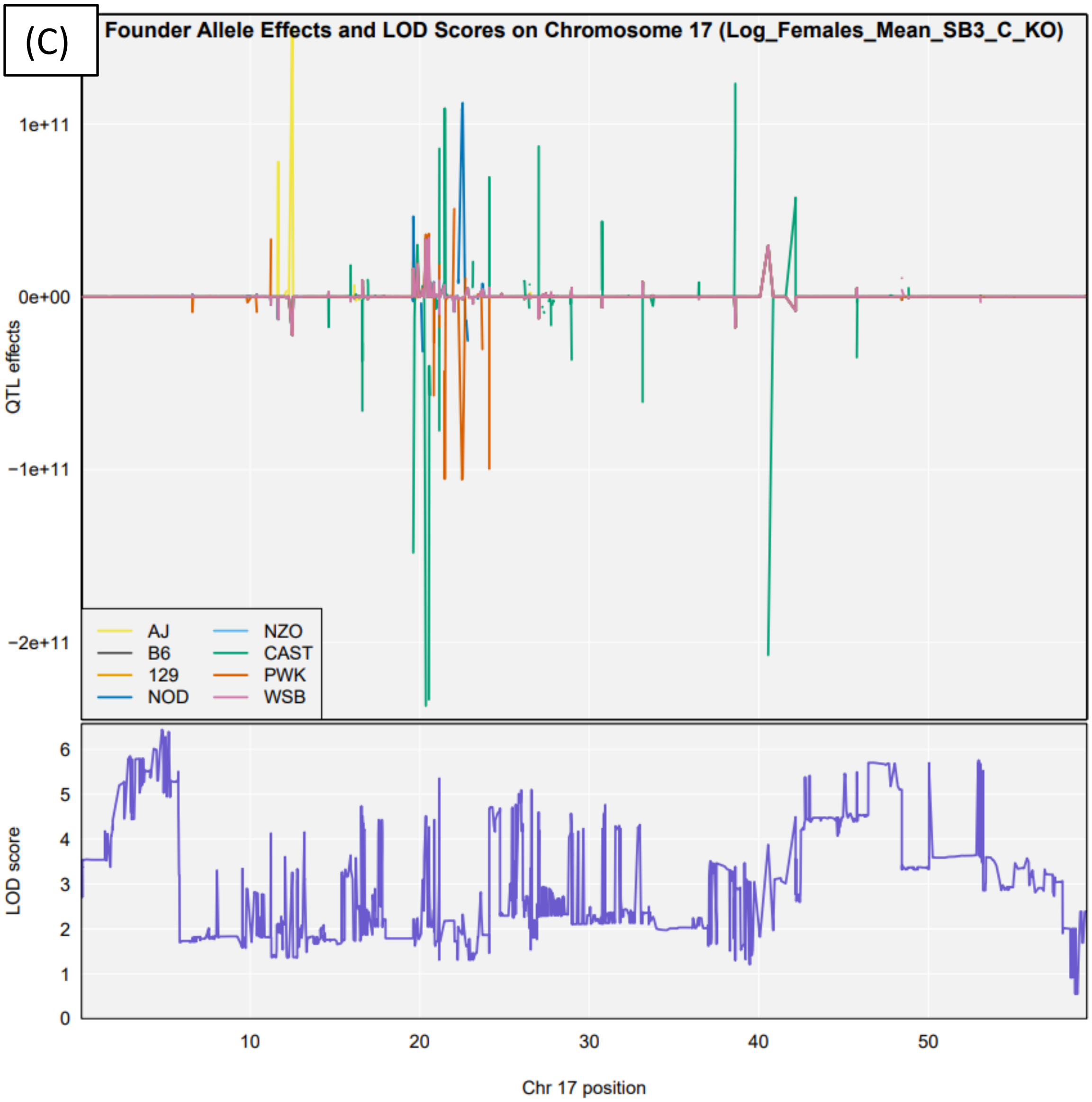
(C)
Founder Allele Effects and LOD Scores on Chromosome 17 (Log_Females_Mean_SB3_C_KO)
QTL effects
1e+11
0e+00
−1e+11
−2e+11
AJ
B6
129
NOD
NZO
CAST
PWK
WSB
LOD score
0
1
2
3
4
5
6
10
20
30
40
50
Chr 17 position

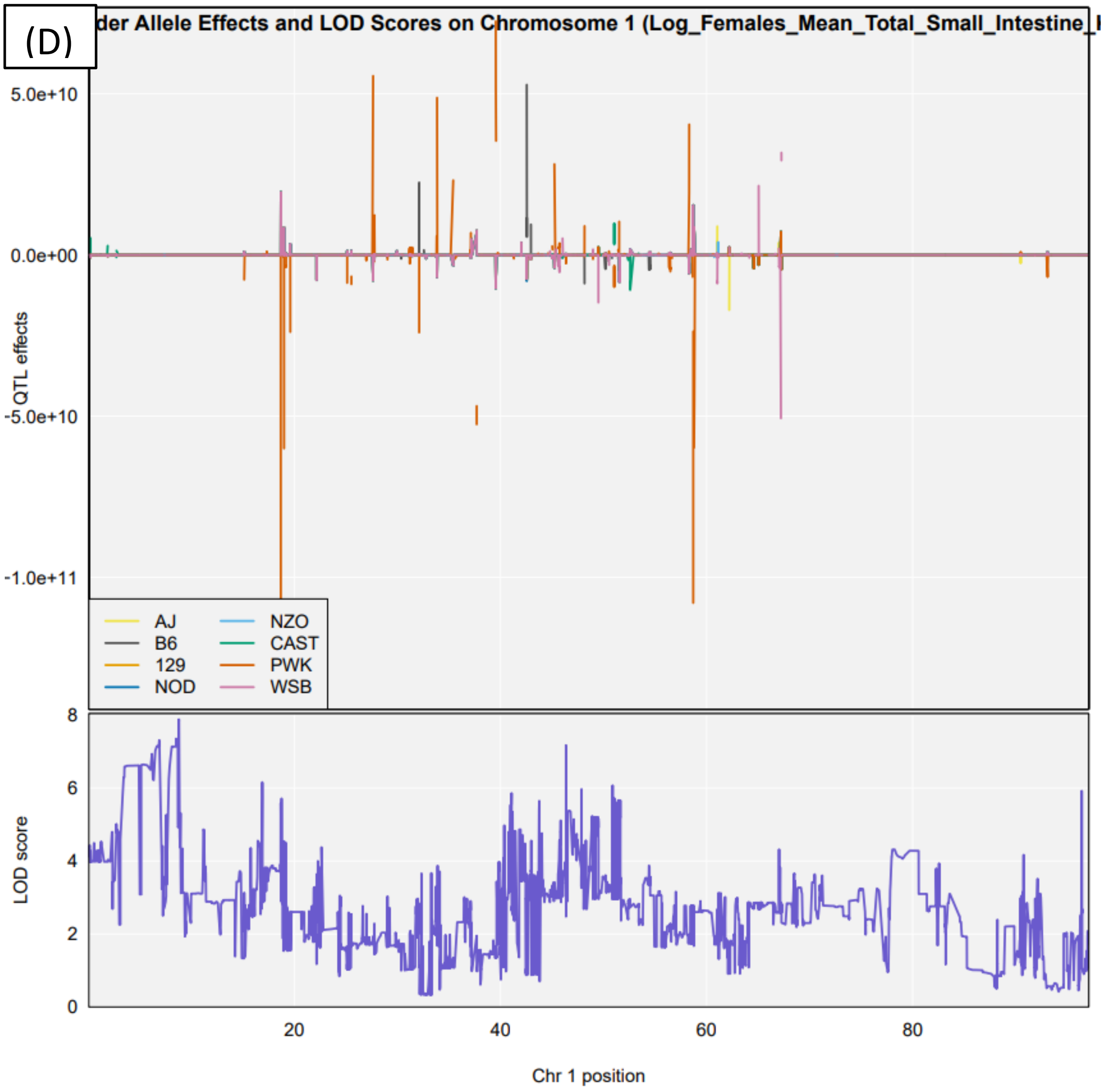
(D)
der Allele Effects and LOD Scores on Chromosome 1 (Log_Females_Mean_Total_Small_Intestine_l
5.0e+10
0.0e+00
-5.0e+10
-1.0e+11
QTL effects
AJ
B6
129
NOD
NZO
CAST
PWK
WSB
8
6
4
2
0
LOD score
20
40
60
80
Chr 1 position
